# The Antitumor Activity of Combinations of Cytotoxic Chemotherapy and Immune Checkpoint Inhibitors Is Model-Dependent

**DOI:** 10.3389/fimmu.2018.02100

**Published:** 2018-10-09

**Authors:** Chloé Grasselly, Morgane Denis, Aurore Bourguignon, Nolan Talhi, Doriane Mathe, Anne Tourette, Laurent Serre, Lars Petter Jordheim, Eva Laure Matera, Charles Dumontet

**Affiliations:** ^1^Anticancer Antibodies, CRCL, INSERM U1052, CNRS UMR 5286, CLB, UCBL, Lyon, France; ^2^ANTINEO, Lyon, France; ^3^Univ Lyon, Université Claude Bernard Lyon 1, INSERM 1052, CNRS 5286, Centre Léon Bérard, Cancer Research Center of Lyon, Lyon, France; ^4^Hospices Civils de Lyon, Lyon, France

**Keywords:** combination therapy, PD-1, PD-L1, chemotherapy, oncology, tumor microenvironment, preclinical mouse models

## Abstract

In spite of impressive response rates in multiple cancer types, immune checkpoint inhibitors (ICIs) are active in only a minority of patients. Alternative strategies currently aim to combine immunotherapies with conventional agents such as cytotoxic chemotherapies. Here, we performed a study of PD-1 or PDL-1 blockade in combination with reference chemotherapies in four fully immunocompetent mouse models of cancer. We analyzed both the *in vivo* antitumor response, and the tumor immune infiltrate 4 days after the first treatment. *in vivo* tumor growth experiments revealed variable responsiveness to ICIs between models. We observed enhanced antitumor effects of the combination of immunotherapy with chemotherapy in the MC38 colon and MB49 bladder models, a lack of response in the 4T1 breast model, and an inhibition of ICIs activity in the MBT-2 bladder model. Flow cytometry analysis of tumor samples showed significant differences in all models between untreated and treated mice. At baseline, all the tumor models studied were predominantly infiltrated with cells harboring an immunosuppressive phenotype. Early alterations of the tumor immune infiltrate after treatment were found to be highly variable. We found that the balance between effector cells and immunosuppressive cells in the tumor microenvironment could be altered with some treatment combinations, but this effect was not always correlated with an impact on *in vivo* tumor growth. These results show that the combination of cytotoxic chemotherapy with ICIs may result in enhanced, similar or reduced antitumor activity, in a model- and regimen-dependent fashion. The present investigations should help to select appropriate combination regimens for ICIs.

## Introduction

Immune checkpoint inhibitors (ICI), such as anti-PD-1 (Programmed cell death 1) or anti-PD-L1 (Programmed death-ligand 1) antibodies, are among the most important recent breakthroughs in oncology. As an example, monoclonal anti-PD-1 and anti-PD-L1 antibodies (mAb) showed impressive efficacy in clinical trials for the treatment of unresectable or metastatic melanoma, metastatic non-small cell lung cancer, renal cancer, and more recently for urothelial carcinoma and Hodgkin's lymphoma (1–7). These monoclonal antibodies block the interaction between PD-1 (Programmed Death 1) molecule, expressed at the surface of T cells and other immune cells, and PD-L1 (Programmed Death-Ligand 1), expressed in multiple types of cancer cells. The PD-1/PD-L1 axis induces an inhibitory signal in T cells, and PD-1/PD-L1 pathway blockade restores T cell function resulting in increased proliferation and cytotoxic activity, subsequently improving anti-tumor immune response (8).

Unfortunately, a non-negligible proportion of patients presents with innate resistance to PD-1/PD-L1 axis blockade, particularly because of lack of expression of PD-L1 by tumor cells or to the immunosuppressive effect of the tumor microenvironment. As a consequence some common cancer types have very low response rates, such as breast and prostate cancer (9, 10). More alarmingly, a significant subpopulation of patients treated with ICI who presented an initial response to therapy will develop acquired resistance, with disease progression after some period of time. Zaretsky et al. highlighted mutations in beta-2-microglobulin, resulting in reduced HLA class I surface expression, as a cause for acquired resistance to anti-PD-1 mAb therapy. In an additional study, they described mutations in JAK1 and JAK2, involving the interferon gamma pathway (10–12). Further hypotheses for this resistance phenotype include genetic alterations (mutations, deletions, epigenetic modifications) which can lead to altered expression of tumor neo-antigens (10). Expression of alternative checkpoints such as LAG-3, TIGIT, TIM-3, and ICOS in the tumor microenvironment may also play a key role regarding clinical outcomes and are currently being explored. Evidence of immune checkpoint expression modulation in association with acquired resistance to anti-PD-1 has been shown by Koyama et al., with an up-regulation of TIM-3 (13).

For this reason, it is crucial to develop new therapeutic approaches to enhance the therapeutic effects of PD-1/PD-L1 blockade, and to avoid resistance phenomena. To this end, efforts are currently engaged including the use of conventional chemotherapies to improve the anti-tumor activity of monoclonal antibodies. Some of these combination strategies are currently studied in various cancer types (14–20). One of the aims of combining chemotherapy with ICIs is to trigger antigen release via the cytotoxic cell death activity of chemotherapy leading to immune stimulation and improving the activity of PD-1 / PD-L1 blocking agents. Moreover, the impact of chemotherapies on the leucocyte composition of the tumor infiltrate might be crucial: as an example, cyclophosphamide has been described to decrease the proportion of regulatory T cells (Treg) and gemcitabine has been outlined to reduce MDSC, two immunosuppressive cell populations usually associated with bad prognosis in cancer (21–23). Several clinical phase I trials combining ICI and chemotherapies are currently ongoing, in particular in NSCLC, with nivolumab in combination with associations of cisplatin and gemcitabine, cisplatin and pemetrexed, or carboplatin and paclitaxel, as well as pembrolizumab in combination with two different chemotherapies (15). Overall, the cytotoxic role of chemotherapy potentially drives immune activation, supporting the study of combinations between ICI and chemotherapies (20, 24, 25).

To explore the possible interaction between conventional regimens and ICIs, we evaluated four preclinical models in order to determine the impact on the *in vivo* efficacy of these combinations, and to analyse the consequences on the tumor microenvironment.

## Results

### The combination of cytotoxic chemotherapy regimens with anti-PD-1 or anti-PD-L1 antibodies impacts differently on anti-tumor activity

We performed an exploratory study of various combination regimens of chemotherapies with immune checkpoint blockers anti-PD-1 and anti-PD-L1, in several murine syngeneic preclinical models. The chemotherapeutic regimens were chosen in order to be similar to those used in the chosen tumor types in patients: capecitabine and oxaliplatin for MC38 colon cancer, methotrexate, vinblastine, doxorubicin and cisplatin (MVAC regimen) for MB49 and MBT-2 bladder cancers, and cyclophosphamide and doxorubicin for 4T1 breast cancer. These combination regimens were first evaluated at different dose levels chosen in order to induce acceptable toxicity (no impact on animal well-being and less than 10% loss of body weight). In some cases however these maximally tolerated chemotherapy regimens did not significantly impact on the tumor growth of established tumors in some models (MC38, MB49, and MBT-2).

**Figure 1:**
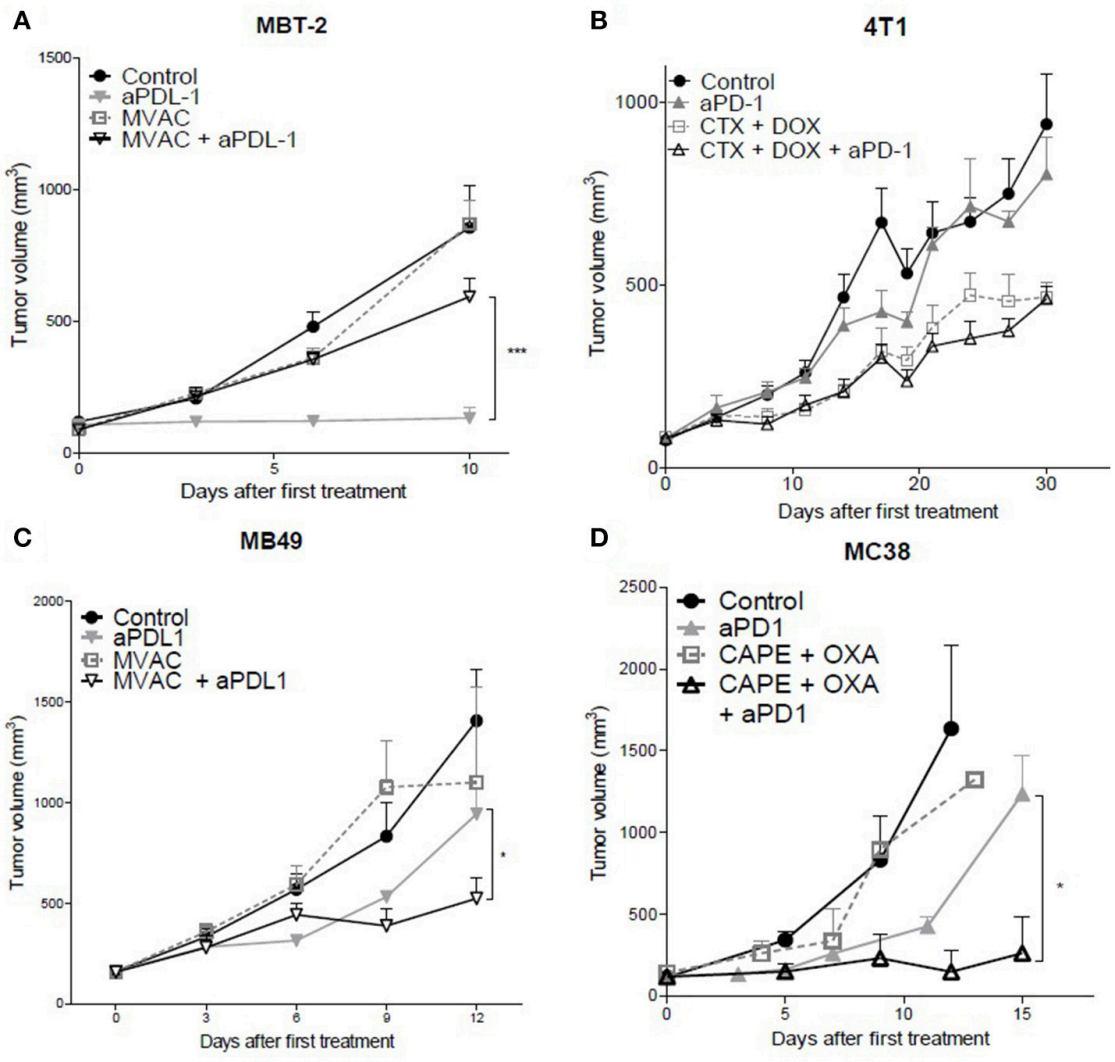
*In vivo* tumor growth in mice treated with ICI alone or in combination with chemotherapy. Anti-PD1 (clone RMP1.14) or anti-PDL1 (clone 10F.9G2) mAb (12,5 mg/kg, ip, q1wk) were administered in combination with **(A,C)** methotrexate (1 mg/kg, ip, q1wk), vinblastine (0.1 mg/kg, ip, q1wk), doxorubicin(1 mg/kg, ip, q1wk) and cisplatin(1 mg/kg,ip, q1wk, MVAC) in SC bladder cancer MB49 and MBT-2, and with **(B)** cyclophosphamide (CTX, 100 mg/kg, ip, q1wk) and doxorubicin (DOX, 2 mg/kg, ip, q1wk) in SC metastatic breast cancer 4T1, and with **(D)** capecitabine (CAPE, 250 mg/kg, po 5 days a week) and oxaliplatin (OXA, 5 mg/kg, ip, q1wk) in SC colorectal cancer MC38. Data shown are mean tumor volumes+ SEM. *n* = 5 to 6 mice/group **(A)**, *n* = 6 mice/group **(B)**, *n* = 6 mice/group **(C)**, *n* = 3 to 6 mice/group **(D)**. *p < 0.05 and ***p < 0.001 using Student's *t*-test.

The combination of the ICI with cytotoxic regimens proved to be more effective than either regimen used separately for the combination of capecitabine/oxaliplatin with anti-PD-1 Mab in the MC38 colorectal cancer model (Figure 1D) as well as with the combination of MVAC with anti-PD-L1 Mab in the MB49 bladder cancer (Figure 1C) while anti-PD-1 in combination with the MVAC regimen did not show any improved activity in the MB49 model (Figure S2B). In the 4T1 breast cancer model, the combination of cyclophosphamide/doxorubicin with anti-PD-1 or anti-PD-L1 Mabs did not show any increased anti-tumor effect (Figures 1B, S2A). Conversely, the combination of MVAC regimen with anti-PD-L1 in the MBT-2 bladder cancer model significantly reduced anti-tumor activity in comparison to single agent anti-PD-L1 (Figure 1A).

These results show that the combination of a cytotoxic regimen with an ICI may have different consequences, including enhanced activity, no impact of the combination, or a reduced anti-tumor activity. Remarkably the MVAC regimen had a different impact when combined with anti-PD-L1 Mab in the MB49 bladder model and in the MBT2 bladder model, supporting an influence of the model on the sensitivity to combination regimens.

### Flow cytometry analysis of tumor immune infiltrate shows a strong model-dependent heterogeneity

**Figure 2:**
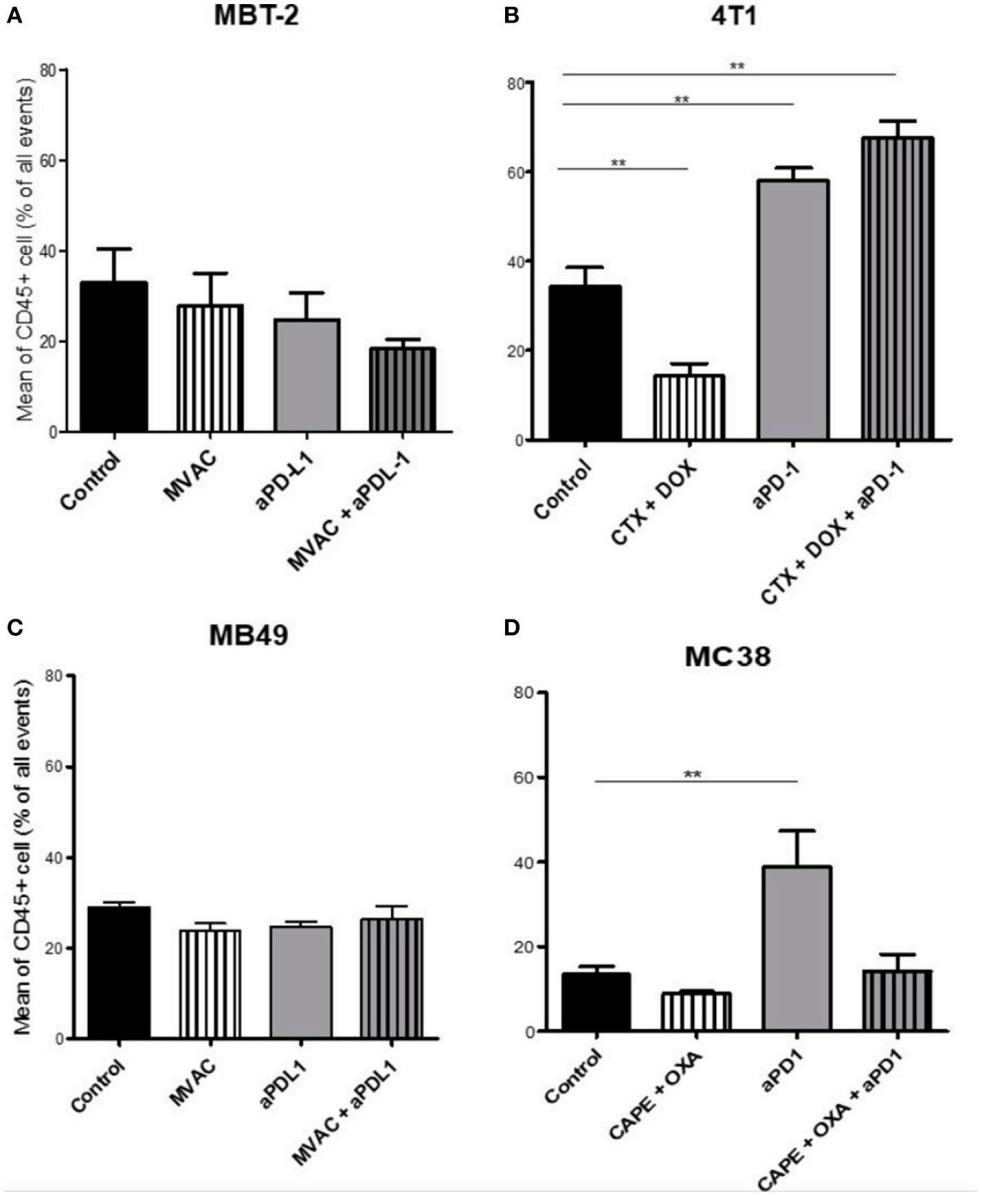
Total leukocyte infiltrate in tumors from mice treated with ICI alone or in combination with chemotherapy. Effect of chemotherapies, anti-PD1 or anti-PDL1 mAbsand their combination on total leucocyte infiltrate in various preclinical tumor models. Flow cytometry analysis of total CD45^+^ cells of MBT-2 tumors **(A)**, 4T1 tumors **(B)**, MB49 **(C)**, and MC38 tumors **(D)**. Mice were treated as in Figure 1. Data are shown as mean values+ SEM. *n* = 5 to 6 mice/group **(A)**, *n* = 5 to 6 mice/group **(B)**, *n* = 6 mice/group **(C)**, *n* = 5 mice/group **(D)**. **p < 0.01 using Mann-Whitney test.

**Figure 3:**
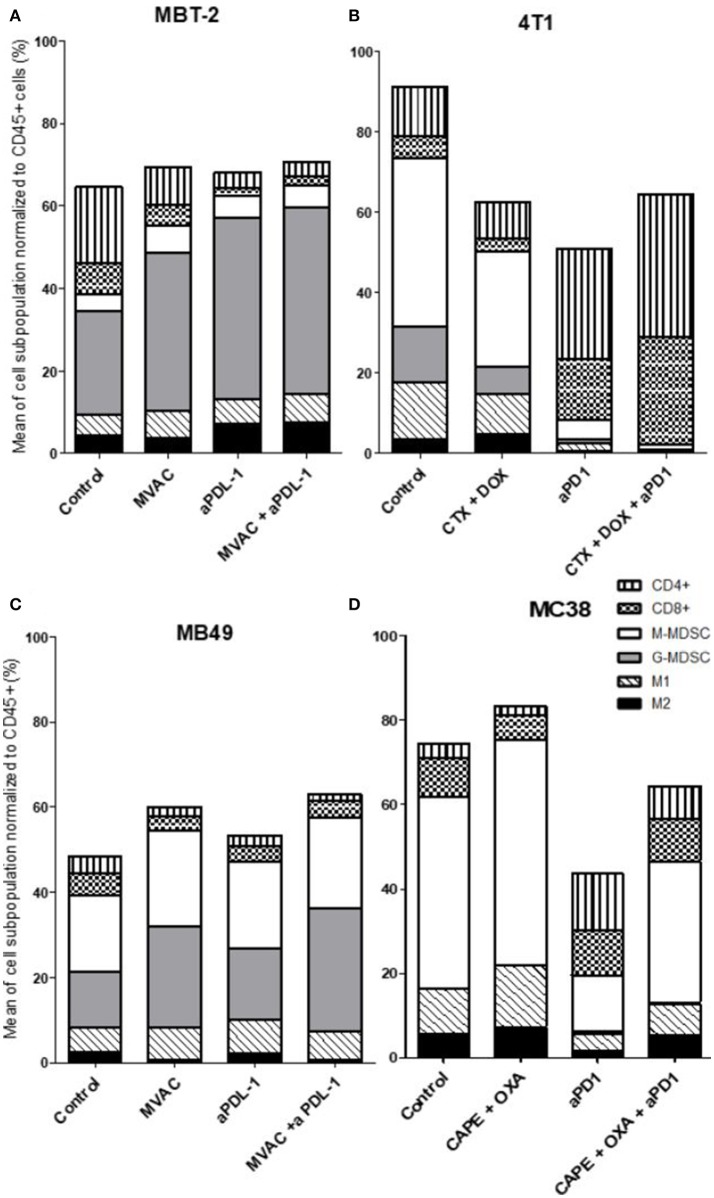
All the preclinical models studied are predominantly infiltrated with cells considered as ≪ immunosuppressive ≫, particularly MDSCs. Effect of chemotherapies, anti-PD1 or anti-PDL1 Mabs and their combination on immune cells subpopulations in various preclinical tumor models. Flow analysis of immune cells subpopulations of total CD45^+^ cells of MBT-2 tumors **(A)**, 4T1 tumors **(B)**, MB49 **(C)**, and MC38 tumors **(D)**. Mice were treated as in Figure 1. Data are shown as Mean of immune cells in each groups normalized from total CD45+ cells, *n* = 5 to 6 mice/group **(A)**, *n* = 5 to 6 mice/group **(B)**, *n* = 6 mice/group **(C)**, *n* = 5 mice/group **(D)**.

**Figure 4:**
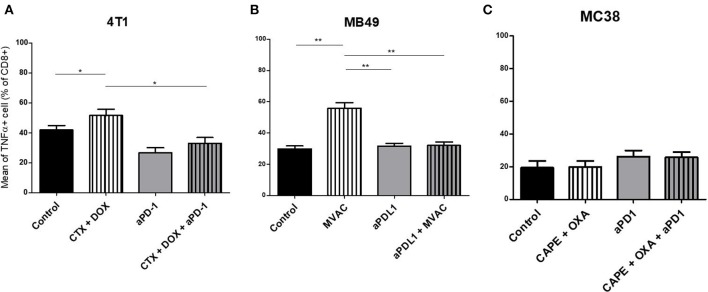
Activated T cells are increased by chemotherapies in breast and bladder cancer. Effect of chemotherapies, anti-PD1 or anti-PDL1 Mabs and their combination on T effector cells activation in various preclinical tumor models. Flow cytometric analysis of TNFα+ CD8+ T cells of total CD45^+^ cells of 4T1 tumors **(A)**, MB49 **(B)**, and MC38 tumors **(C)**. Mice were treated as in Figure 1. Data are shown as Mean + SEM, *n* = 5 to 6 mice/group **(A)**, *n* = 6 mice/group **(B)**, *n* = 5 mice/group **(C)**, Mann–Whitney test: *P < 0.05 and **P < 0.01. Results not shown for MBT-2 bladder cancer (Figure S4).

**Figure 5:**
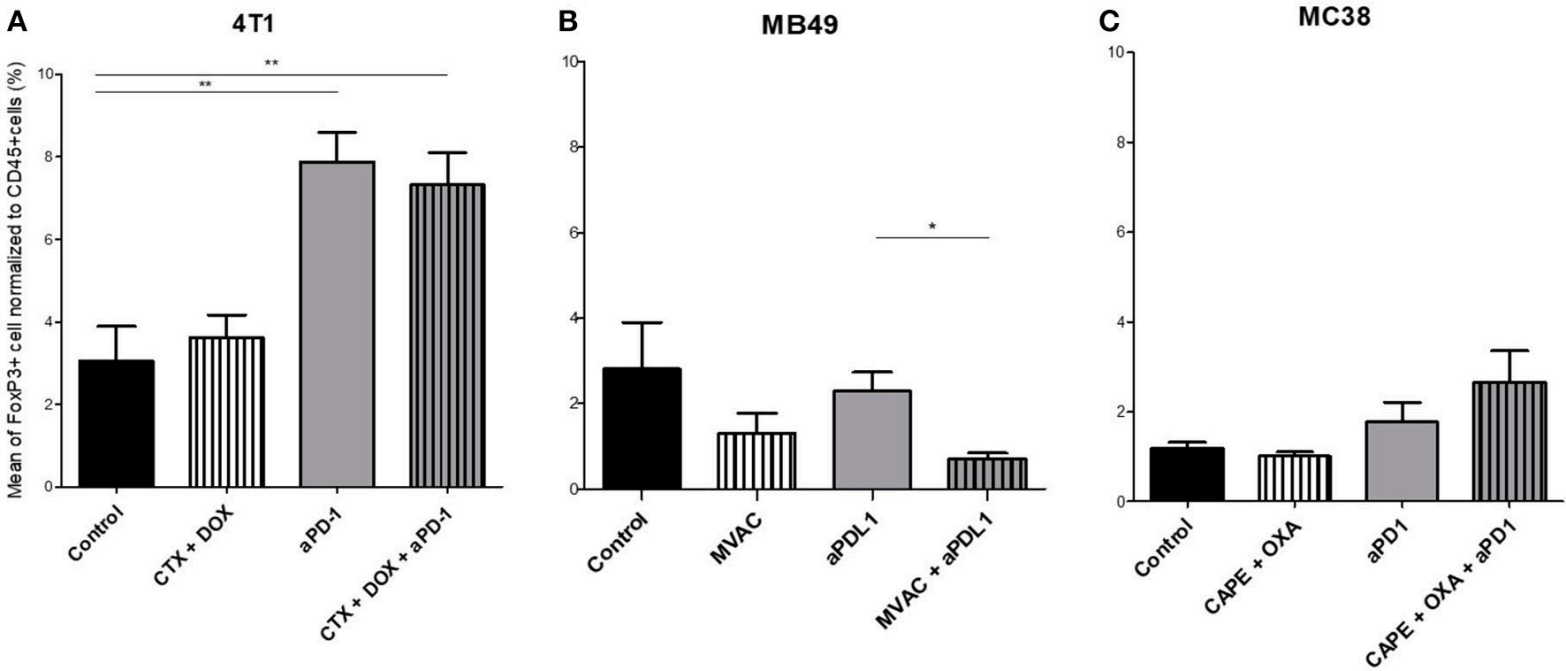
Combination of MVAC with anti-PDL1 significantly reduces T reg cells infiltrate compared with anti-PDL1 single treatment in bladder cancer MB49. Effect of chemotherapies, anti-PD1 or anti-PDL1 Mabs and their combination on T regulator cells in various preclinical tumor models. Flow cytometric analysis of FoxP3+ CD4 T cells of total CD45^+^ cells of 4T1 tumors **(A)**, MB49 **(B)**, and MC38 tumors **(C)**. Mice were treated as in Figure 1. Data are shown as Mean + SEM, *n* = 5 to 6 mice/group **(A)**, *n* = 6 mice/group **(B)**, *n* = 5 mice/group **(C)**, Mann–Whitney test: *P < 0.05 and **P < 0.01. Results not shown for MBT-2 bladder cancer (Figure S4).

We characterized the tumor immune infiltrate profiles by flow cytometry for each tumor type and for each treatment condition, 4 days after the first administration of treatment (Table S2, Figures S1, S3). This included total immune infiltrate (Figure 2), effector cells such as CD4+ T cells, CD8+ T cells, Type 1 macrophages (Figure 3) and activated CD8+ T cells with TNFα+ cells (Figure 4). Additionally, we quantified ≪ pro-tumor ≫ cells including Granulocytic and Monocytic Myeloid Derived Suppressor Cells (respectively G-MDSC and M-MDSC), Type 2 macrophages (Figure 3) and T reg cells (Figure 5). The following results are expressed as the mean of the percentages of cells in each treatment group, normalized to total CD45+ cells (except for total leucocyte infiltrate which is expressed as a percentage of all events, and for TNFα+ cells which is presented as the percentage of CD8+ T cells). Individual data are shown on Figure S5.

#### Total leukocyte infiltrate

The baseline total leucocyte infiltrate, evaluated by CD45 labeling, was similar in three of the models from untreated mice with values of 36 % in 4T1, 33% in MBT2, 29% in MB49, but was lower in MC38 with a lower baseline level of 13 % (Figure 2). Among the chemotherapy regimens used only doxorubicin/cyclophosphamide induced a significant decrease of the total leukocyte infiltrate while MVAC and capecitabine/oxaliplatin had no significant impact. Anti-PD-1 therapy was associated with an increase of the total infiltrate in the MC38 and 4T1 models while anti-PD-L1 therapy had no impact. Furthermore this increase was impeded by the combination with capecitabine/oxaliplatin in the MC38 model but not by doxorubicin/cyclophosphamide in the 4T1 model.

#### Immune populations

We observed baseline differences in the percentages of CD4 and CD8 infiltrates as well as in the percentages of M1 macrophages. MDSCs were abundant in all models studied but with some remarkable differences between models. G-MDSCs were abundant in MBT-2 tumors but absent in MC38 tumors. M-MDSCs were present in all tumors and represented almost half of the immune infiltrate in MC38 tumors, as previously described (Figure 3) (26). Macrophages were present at baseline in all tumor types, representing 8 to 18 % of all immune cells with a relative preponderance of M1 macrophages in comparison with M2 cells (ratios of 1.5 to 4 depending on tumor model).

Several alterations in immune populations were observed in response to a single administration of therapy. These include a decrease of the CD4+ and CD8+ T cell infiltrates with the different chemotherapy regimens used and a strong decrease of MDSCs after treatment with anti-PD-1 antibodies but not with anti-PD-L1 antibodies. T cell infiltrates were increased after exposure to chemotherapy + anti-PD-1, to a stronger extent in the 4T1 model than in the MC38 model. Conversely T cells tended to be quantitatively reduced in mice exposed to chemotherapy + anti-PD-L1. Macrophage infiltrates (both M1 and M2) were reduced after exposure to anti-PD-1 but not to anti-PD-L1 antibodies. The analysis of activated CD8 cells (Figure 4) found a significant difference in 4T1 and MB49 bearing mice exposed to chemotherapies, with an increase of the activated cells in these groups. Treg cells were found to be significantly altered in MB49 bearing mice after exposure to anti-PDL-1 + MVAC, and an increase in 4T1 bearing mice exposed to anti-PD-1 alone or in combination with chemotherapy was observed (Figure 5).

Overall our observations reveal strong differences between models in the relative proportions of immune cell populations at the basal level as well as in variations to therapy which appear to be both dependent on the model and on the treatment administered.

### Effect of therapy on immune checkpoint expression

**Figure 6:**
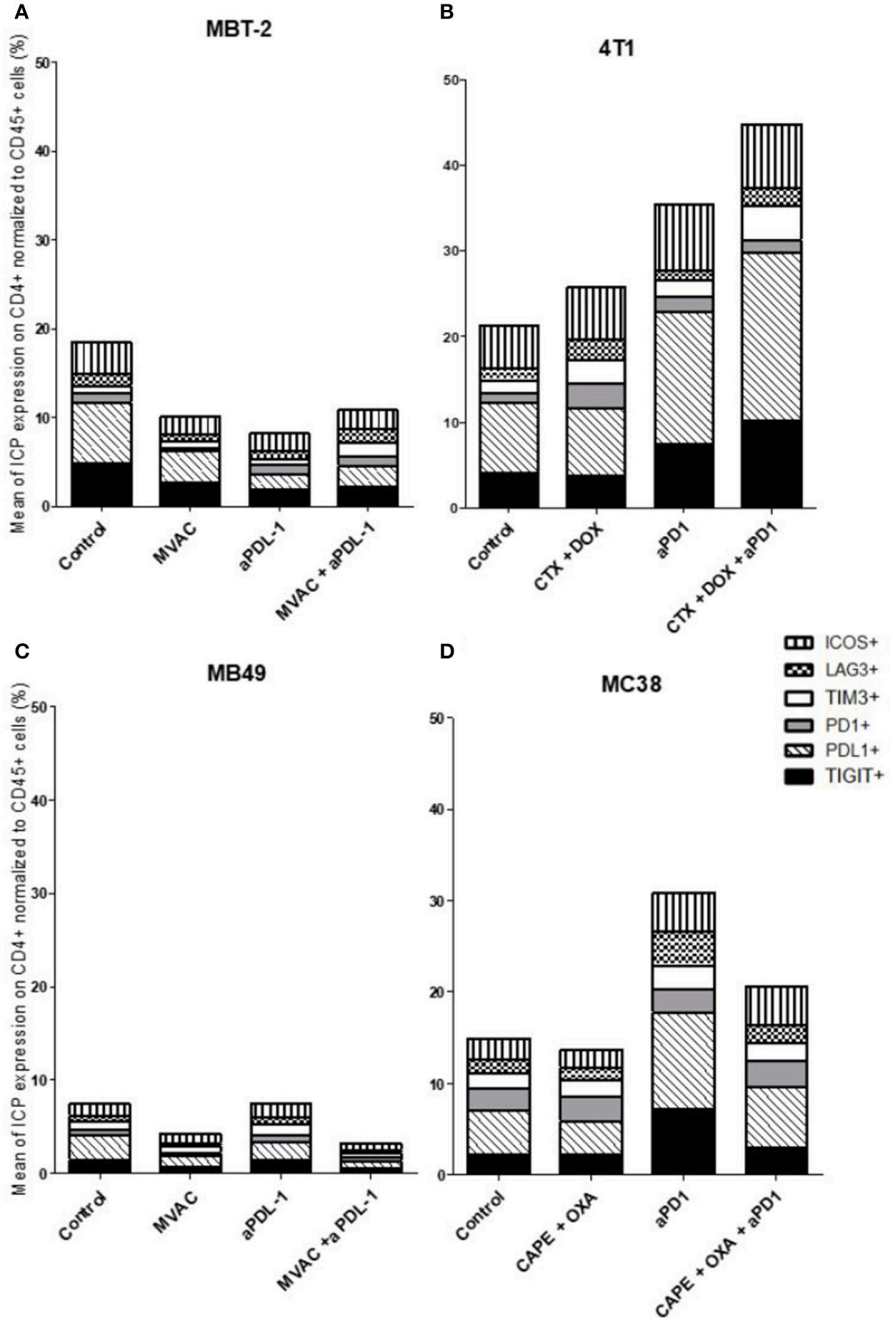
Combination of cyclophosphamide and doxorubicine increase inhibitory checkpoint on immune CD4+ T cells in 4T1 breast cancer model. Effect of chemotherapies, anti-PD1 or anti-PDL1 Mabs and their combination on alternative immune checkpoints expression on CD4 T cells in various preclinical tumor models. Flow cytometric analysis of CD279 (PD-1), CD223 (LAG-3), TIM-3, CD278 (ICOS), CD274 (PDL-1), TIGIT on CD4 T cells infiltrate of total CD45^+^ cells of MBT-2 tumors **(A)**, 4T1 tumors **(B)**, MB49 **(C)** and MC38 tumors **(D)**. Mice were treated as in Figure 1. Data are shown as Mean percentage of CD4 T cells which express the different immune checkpoint, *n* = 5 to 6 mice/group **(A)**, *n* = 5 to 6 mice/group **(B)**, *n* = 6 mice/group **(C)**, *n* = 5 mice/group **(D)**.

**Figure 7:**
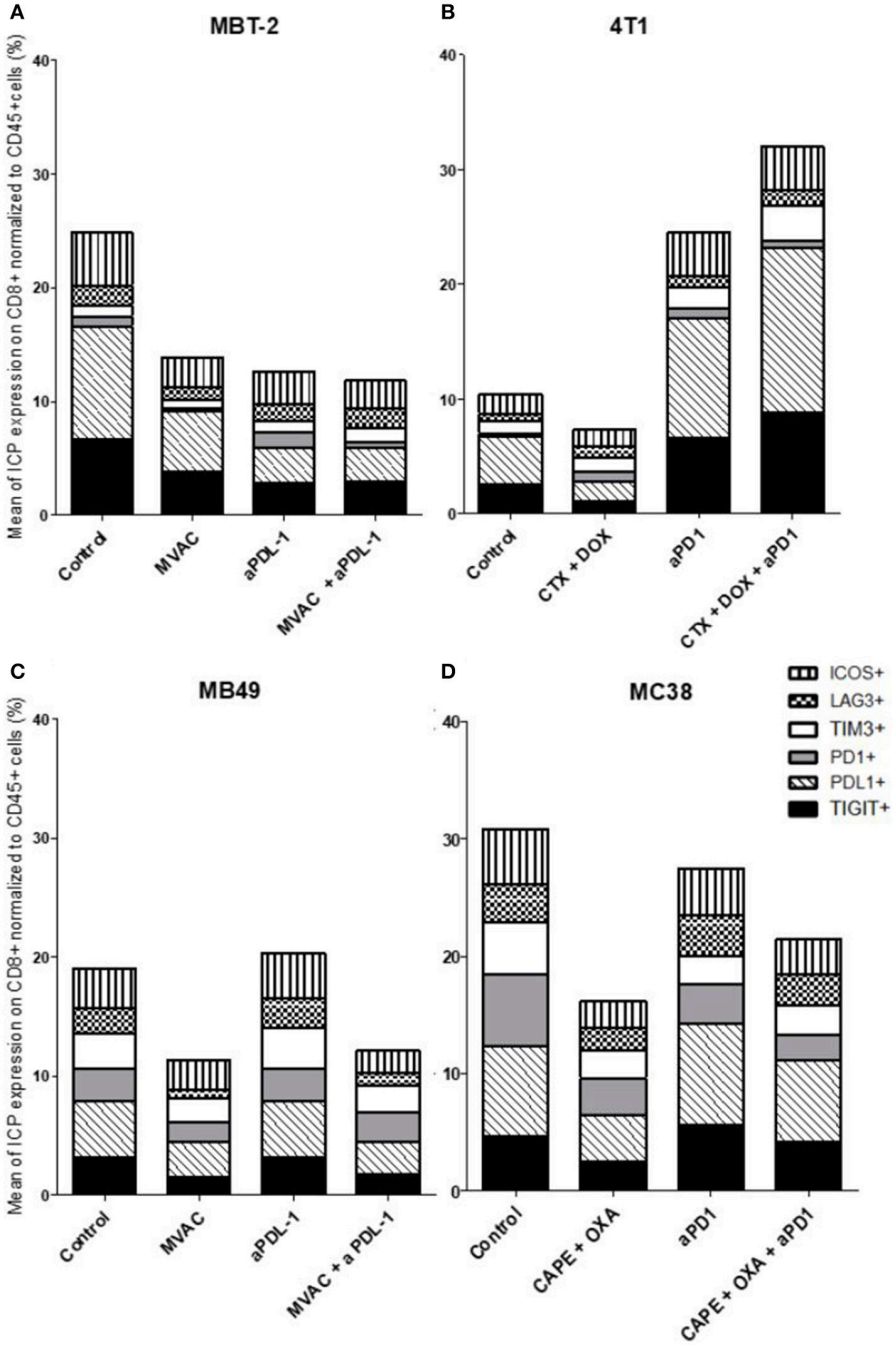
Chemotherapies decrease inhibitory checkpoint expression on immune CD8+ T cells. Effect of chemotherapies, anti-PD1 or anti-PDL1 Mabs and their combination on alternative immune checkpoints expression on CD8+ T cells in various preclinical tumor models. Flow cytometric analysis of CD279 (PD-1), CD223 (LAG-3), TIM-3, CD278 (ICOS), CD274 (PDL-1), TIGIT on CD8 T cells infiltrate of total CD45^+^ cells of MBT-2 tumors **(A)**, 4T1 tumors **(B)**, MB49 **(C)**, and MC38 tumors **(D)**. Mice were treated as in Figure 1. Data are shown as Mean percentage of CD8+ T cells which express the different immune checkpoint, *n* = 5 to 6 mice/group **(A)**, *n* = 5 to 6 mice/group **(B)**, *n* = 6 mice/group **(C)**, *n* = 5 mice/group **(D)**.

**Figure 8:**
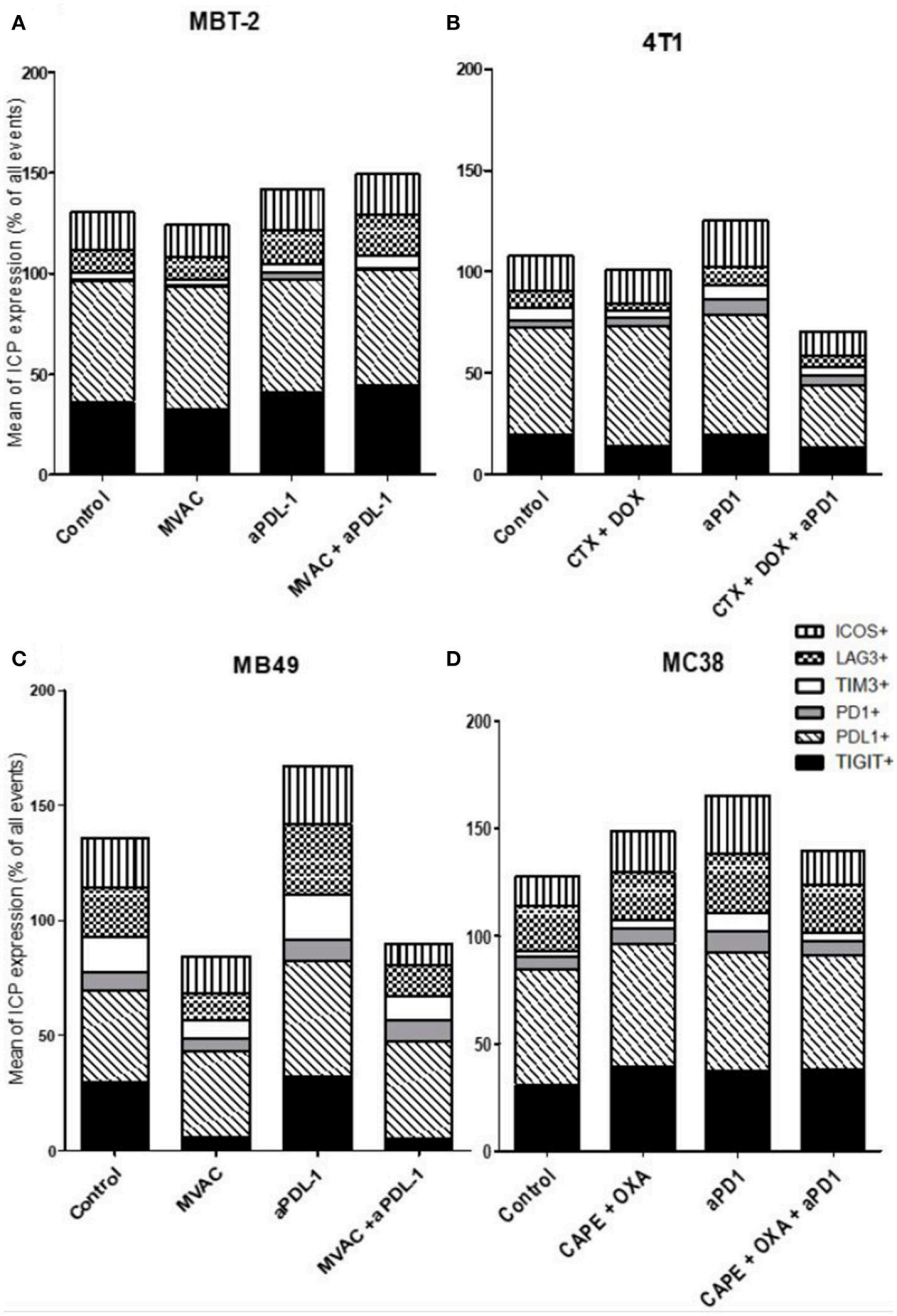
MVAC chemotherapy regimen induces a large decrease of TIGIT expression on tumor cells in MB49 bladder cancer. Effect of chemotherapies, anti-PD1 or anti-PDL1 Mabs and their combination on alternative immune checkpoints expression on tumor cells in various preclinical tumor models. Flow cytometric analysis of CD279 (PD-1), CD223 (LAG-3), TIM-3, CD278 (ICOS), CD274 (PDL-1), TIGIT on tumor cells of total CD45^+^ cells of MBT-2 tumors **(A)**, 4T1 tumors **(B)**, MB49 **(C)**, and MC38 tumors **(D)**. Mice were treated as in Figure 1. Data are shown as Mean percentage of tumor cells which express the different immune checkpoint, *n* = 5 to 6 mice/group **(A)**, *n* = 5 to 6 mice/group **(B)**, *n* = 6 mice/group **(C)**, *n* = 5 mice/group **(D)**.

In parallel to intra-tumor T cell and immunosuppressive cell quantifications, we used our mouse models of combination regimens to investigate the expression of T cell exhaustion markers such as the T-cell immunoglobulin and mucin-domain containing-3 (TIM-3), the Lymphocyte-activation gene 3 (LAG3) and the T cell immunoreceptor with Ig and ITIM domains (TIGIT). We also evaluated PD-1 expression on the T cell surface. In addition, we also looked at the T cell activation marker, Inducible T-cell costimulator (ICOS). Indeed, some studies already described upregulation of TIM-3 as a mechanism of adaptive resistance to anti-PD-1 therapy (27). Therefore, we sought to evaluate the impact of chemotherapies and combination of chemotherapies in addition to PD-1/PD-L1 axis disruption on alternative checkpoint expression. In this way, we analyzed these checkpoints in CD4+ and CD8+ T cells (Figures 6, 7), as well as their expression on tumor cells (Figure 8). Individual data are shown in Figures S6–S8.

In the case of CD4+ T cells (Figure 6), we found a strong increase of TIGIT and ICOS expression in response to single agent anti-PD-1 antibody, with mitigation of this effect on ICOS by combination with capecitabine/oxaliplatin but not doxorubicin/cyclophosphamide. MVAC tended to decrease the expression of the other immune checkpoints on CD4 cells, with no mitigating effect of PD-L1 antibody. In the case of CD8+ T cells (Figure 7), TIGIT and ICOS were increased in response to anti-PD-1 antibody in the 4T1 model and to a lesser extent in the MC38 model. MVAC reduced immune checkpoint levels similarly to what was observed in CD4+ T cells. Anti-PD-L1 antibodies had no effect on CD8 cells in the MB49 model while tending to reduce immune checkpoint expression in the MBT2 model.

Regarding immune checkpoint expression on tumor cells (Figure 8), we did not observe a strong modulation of their expression with anti-PD-1, anti-PD-L1 or the combinations in most cases (Figure 8D). However, we noticed a remarkable decrease of TIGIT expression on tumor cells after exposure to the MVAC chemotherapy regimen in the MB49 model (Figures 8C, S8). Interestingly, we did not find a similar collapse after MVAC exposure in the MBT-2 model (Figure 8A). Expression of PD-L1 on tumor cells has been reported to be correlated with improved survival and response rate in the context of anti-PD-1 and anti-PD-L1 trials, and has been suggested as a predictive biomarker (28). In our models treatments did not modify PD-L1 expression in tumor cells except for the combination cyclophosphamide/doxorubicin + anti-PD-1 in the 4T1 model (Figure 8B). On the contrary, we observed an increase of PD-L1 expression on tumor cells when anti-PD-L1 monotherapy was used in the bladder cancer model MB49 (Figure 8C).

## Discussion

Immune checkpoint blockade is one of the most promising therapies for a wide range of cancer types. Approved therapies such as anti-PD-1 and anti-PD-L1 induce impressive responses in advanced disease and considerably improve survival in some patients. However, a non-negligible proportion of patients do not respond to these therapies. Consequently, there is an important need to find new therapeutic approaches to improve response rates. Combinations with standard regimens such as cytotoxic chemotherapy is a logical approach but raises a number of possible issues (29). As exemplified by our results combinations may have unexpected effects ranging from enhanced to reduced anti-tumor activity in comparison to single-agent therapies. We observed an improved anti-tumor activity of chemotherapies in combination with ICI in colorectal cancer and one bladder cancer model (MB49]. However, in the context of a metastatic breast cancer model, chemotherapy in combination with anti-PD-1 did not show any anti-tumor efficacy. In the worst-case scenario, chemotherapy had a negative impact on tumor growth, such as we observed in another model of bladder cancer (MBT-2).

There are potentially several mechanisms through which chemotherapeutic regimens and ICIs could interact and modulate anti-tumor activity. While cytotoxic agents cause cell death by a direct effect on tumor cells, they are also likely to enhance tumor cell death indirectly by the process of immunogenic cell death, which might secondarily facilitate the anti-tumor activity of ICIs (30). As an example, cyclophosphamide (used in the 4T1 model) appears to induce tumor cell death in a preclinical model of lymphoma, and oxaliplatin increases tumor infiltration, notably by CD8+ T cells, in a preclinical prostate cancer (31, 32). Alternatively some cytotoxic agents are known to be lymphotoxic, with potential deleterious effects when acting on anti-tumor T cells or beneficial effects when acting on pro-tumor T cells such as Tregs. It is likely that cytotoxic agents could act on other immune populations as well. Additionnally cytotoxic agents have shown to prime tumor cells for granzyme-mediated death (33).

Two of our preclinical models showed enhanced activity of the combination of cytotoxic agents and ICIs, the capecitabine/oxaliplatin regimen combined with anti-PD-1 antibodies in the MC38 model and the MVAC regimen combined with anti-PD-L1 antibodies in the MB49 model. In the MC38 model basal immune cells infiltration was relatively low and was increased after treatment with anti-PD-1 antibodies but this effect was mitigated in the combination regimen. Additionnally MC38 tumors had a strong content in M-MDSCs which was decreased by anti-PD-1 antibodies while the CD4+ and CD8+ T cell infiltrates were proportionally increased. In the MB49 model the most remarkable modification induced by the therapeutic combination was a modification in the proportion of MDSCs and an increase of the M1/M2 macrophages ratio from 2.5 to 9 in the combination group. Remarkably in the MC38 model, ICOS expression was increased and inhibitory checkpoint expression decreased on CD4 T cells by the combination of anti-PD-1 with capecitabine/oxaliplatin. In the MB49 model inhibitory checkpoints were decreased both on CD4+ and CD8+ T cells after exposure to the cytotoxic regimen alone and to the combination regimen. These results suggest that modulation of immune checkpoints on immune cells by cytotoxic agents could impact on the potency of ICIs when these are co-administered.

We observed no benefit of the combination in the 4T1 model exposed to doxorubicin/cyclophosphamide and anti-PD-1 antibodies and an antagonistic effect in the MBT2 model exposed to MVAC and anti-PD-L1 antibodies. This result was unexpected in the 4T1 model as the combination profoundly modified the immune microenvironment with a strong decrease in MDSCs and a large increase in the CD4+ and CD8+ T cell infiltrates, including activated CD8+ T cells and increased Tregs. However it should be emphasized that under our treatment conditions 4T1 tumors were not sensitive to single agent anti-PD-1 suggesting that the immune alterations observed were not sufficient to induce a response to these antibodies in the combination regimen. In the MBT2 model we also observed profound alterations in the microenvironment, including a decrease in CD4+ and CD8+ T cells as well as a strong increase of G-MDSCs and M2 macrophages. As MBT2 was sensitive to anti-PD-L1 antibodies but not to the MVAC regimen we hypothesize that in this case the cytotoxic agents were involved in the antagonistic effect observed. These results suggest that the same regimen, in this case MVAC, can have different consequences on the immune microenvironment depending on the baseline situation in the tumor.

There are a number of limitations to our study. The four models studied were implanted in three different strains of mice which may react differently at a systemic level to the therapeutic agents used. In spite of preliminary dose-ranging studies we were not always able to define a cytotoxic regimen which was active in certain models. Moreover, tumor models were established from tumor cells by subcutaneous injection, for homogeneity purpose. Variations between subcutaneous and orthotopic models has already been discussed in the literature (34, 35), and it seems to be relevant to test the combination of chemotherapies with ICIs on tumors grafted into their normal place of development.

It is important to note that different mice strains may have different immune status and thus may display different immune infiltrates in tumors. This aspect has already been shown by Sellers and al., with immune variations specific to the strain (36). However, the diversity of mice immune infiltration can also be considered to reflect the important heterogeneity observed in patients.

Additionally, immunophenotypic analyses were performed at a single time point (day 4 post-therapy) which did not allow us to compare kinetic modifications of the microenvironment in the tumor. Another key parameter is the therapeutic sequence of the cytotoxic agents and the immune checkpoint inhibitors. We chose to initiate both types of treatment simultaneously but it is likely that sequenced administration could lead to different results.

Overall our results emphasize the heterogeneity in the tumor microenvironment of syngeneic models and the diversity of response elicited by treatment combinations. While additional studies combining single agents with ICIs are required to better understand potential interactions, most ongoing clinical trials are evaluating the association of ICIs with combination chemotherapy regimens (Table S3). These trials will provide important data concerning both antitumor activity and auto-immune toxicity.

## Materials and methods

### Cell culture

Murine colon cancer MC38 cells (Kerafast, USA) were cultured in DMEM medium with 10% fetal bovine serum, 100 U/mL penicillin and streptomycin. The murine metastatic breast cancer cell line 4T1 (ATCC, ATCC® CRL-2539™) and the murine bladder cancer cell lines MBT-2 and MB49 (kindly provided by Alain Bergeron, Laval University) were cultured with RPMI medium with 10% fetal bovine serum, 100 U/mL penicillin and streptomycin. Cells were incubated in humidified incubator with 5% CO_2_ at 37°C.

### Establishment and measure of syngeneic SC tumors

MC38 cells and MB49 cells were injected in C57BL6 mice (Janvier Lab), 4T1 cells were injected in BALB/c mice (Charles River Lab) and MBT-2 cells were injected in C3H mice (Charles River Lab). In all cases, suspensions of exponentially growing cancer cells diluted in 0.2 mL of PBS were injected subcutaneously into the right flank of mice (2.10^6^ cells for MC38, MB49 and MBT-2 models, 1.10^6^ cells for the 4T1 model). When tumor volume reached 200 mm^3^ for the MC38, MB49 and MBT-2 models, and 100 mm^3^ for the 4T1 model, mice were randomized and the first treatment was administered (*n* = 8 to 12 mice per group, either *n* = 3 to 6 for tumor growth and *n* = 5 to 6 for flow cytometry analysis). The tumor volume was measured every 3 days (length x width) with a caliper. The tumor volume was determined using the formula: 4/3 × π × r^3^. All mice were raised in SPF environment with free access to standard food and water. This study was approved by the Animal Ethics Committee of the University Claude Bernard of Lyon.

Four days after first administration of treatment, half of the mice in each group were sacrificed to analyze their tumor microenvironment by flow cytometry, while the other mice pursued treatment in order to determine sensitivity to therapy.

### Treatment

The combination regimen used for the MC38 colon adenocarcinoma mouse model was capecitabine (Actavis Group PTC ehf) administered per os 5 days a week at a dose of 250mg/kg and oxaliplatin (Mylan), injected i.p. once a week at a dose of 5 mg/kg + anti-PD-1 (RMP1-14, BioXCell), injected i.p. once a week at a dose of 12.5 mg/kg. For the metastatic 4T1 breast cancer mouse model, the combination was cyclophosphamide (Baxter) injected i.p. once a week at a dose of 100 mg/kg + doxorubicin (Accord Healthcare) injected i.p. once a week at a dose of 2 mg/kg + anti-PD-1 (RMP1-14, BioXCell) or anti-PD-L1 (10F.9G2, BioXCell), injected i.p. once a week at a dose of 12.5 mg/kg. For bladder cancer mouse models, the MVAC regimen consisted in a combination of methotrexate (Mylan) injected i.p. once a week at a dose of 1 mg/kg + vinblastine (EG Labo) injected i.p. once a week at a dose of 0.1 mg/kg + doxorubicin (Accord Healthcare) injected i.p. once a week at a dose of 1 mg/kg + cisplatine (Mylan) injected i.p. once a week at a dose of 1 mg/kg, + anti-PD-L1 (10F.9G2, BioXCell) injected i.p. once a week at a dose of 12.5 mg/kg and + anti-PD-1 (RMP1-14, BioXCell) injected i.p. once a week at a dose of 12.5 mg/kg for MB49 mouse model.

### Flow cytometry

Flow cytometry was used to analyze the tumor immune microenvironment. All tumor tissue samples per groups were collected, dilacerated, counted and cells surface were stained with the following fluorescently conjugated antibodies: anti-CD45 (30F11, BD Biosciences), anti-CD4 (RM4-5, Miltenyi Biotec), anti-CD8 (53-6.7, Miltenyi Biotec), anti-CD161 (PK136, Miltenyi Biotec), anti-CD3 (REA606, Miltenyi Biotec), anti-CD68 (FA-11, Miltenyi Biotec), anti-CD206 (C068C2, BioLegend) anti-CD11b (REA592, Miltenyi Biotec), anti-Ly-6C (1G7.G10, Miltenyi Biotec), anti-Ly6G (REA526, Miltenyi Biotec), anti-PD-1 (HA2-7B1, Miltenyi Biotec), anti-LAG3 (C9B7W, Miltenyi Biotec), anti-TIM3 (REA602, Miltenyi Biotec), anti-ICOS (REA192, Miltenyi Biotec), anti-PD-L1 (10F.9G2, BioLegend), anti-TIGIT (REA536, Miltenyi Biotec), anti-TNFα (REA636, Miltenyi Biotec), anti-FoxP3 (3G3, Miltenyi Biotec). After surface staining, cells were fixed and permeabilized using BD Cytofix/Cytoperm kit and then labeled with FoxP3 and TNFα. Flow cytometry data were acquired on the LSRII flow cytometer, FlowJo software was used for analyses and GraphPad Prism software was used for statistical analysis (Unpaired t test). Isotype controls were from Miltenyi Biotec. Gating schemes are shown on Figures S4, S5.

## Author contributions

CG designed and performed the experiments, derived the models and analyzed the data. LS advised with methods and theory of experiments. MD, AB, EM, and NT assisted CG with experiments. MD and AB analyzed data. AT and DM conceived initial experiments in the lab. CG wrote the manuscript in consultation with CD, LJ, and CD supervised the project.

### Conflict of interest statement

The authors declare that the research was conducted in the absence of any commercial or financial relationships that could be construed as a potential conflict of interest.

## Supplementary material

Figure S1 Experimental design of combination experiments.

Figure S2 Effect of anti-PD1 (clone RMP1.14) or anti-PDL1 (clone 10F.9G2) antibody, 12,5 mg/kg, i.p., q1wk, in combination with cyclophosphamide (CTX) 100 mg/kg, i.p, q1wk and doxorubicin (DOX) 2 mg/kg, i.p, q1wk in SC metastatic breast cancer 4T1 **(A)**, methotrexate 1mg/kg, i.p, q1wk and vinblastine 0,1 mg/kg, i.p, q1wk and doxorubicin 1 mg/kg, i.p, q1wk and cisplatin 1 mg/kg i.p, q1wk (MVAC) in SC bladder cancer MB49 **(B)**, Data are shown as Mean + SEM, *n* = 5 to 6 **(A)**, *n* = 6 **(B)**.

Figure S3 Example of gating strategy for immune cell infiltrate study. Example of flow cytometry analysis results: comparison between Control tumor and tumor treated with cyclophosphamide 100 mg/kg i.p and doxorubicin 2 mg/kg i.p and anti-PD1 12.5 mg/kg i.p. (CTX + DOX + PD1) in SC breast cancer 4T1.

Figure S4 Effect of chemotherapies, anti-PDL1 Mab and their combination on T reg cells **(A)** and T effector cells activation **(B)** in MBT-2 preclinical tumor model. Flow cytometric analysis of TNFα+ CD8+ T cells of total CD45^+^ cells **(A)**, FoxP3+ CD4+ T cells of total CD45+ cells **(B)**. Mice were treated as in Table S1. Data are shown as Mean + SEM, *n* = 5 to 6, Mann–Whitney test: ^*^*P* < 0.05.

Figure S5 Effect of chemotherapies, anti-PD1 or anti-PDL1 mAbs and their combination on leucocyte infiltrate subpopulations in MBT-2, 4T1, MB49, and MC38 preclinical tumor models. Flow cytometric analysis of total CD4+ T cells: CD45+CD3+CD4^+^ **(A)**, CD8+ T cells CD45+ CD3+CD8^+^ **(B)**,Granulocytic Myeloid Derived Suppressor Cells (G-MDSC): CD45+ CD3- CD11b+ Ly6G+ Ly6C- **(C)** Monocytic Myeloid Derived Suppressor Cells (M-MDSC): CD45+ CD3- CD11b+ Ly6G- Ly6C+ (**D**), M1 macrophages: CD45+ CD3- CD11b+ CD68+ CD206- **(E)**), M2 macrophages: CD45+ CD3- CD11b+ CD68- CD206+ **(F)**. Mice were treated as in Table S1. Data are shown as mean values+ SEM, *n* = 5 to 6 mice/group (**A**), *n* = 5 to 6 mice/group (**B**), *n* = 6 mice/group **(C)**, *n* = 5 mice/group **(D)**. ^*^*p* < 0.05 and ^**^*p* < 0.01 using Mann-Whitney test.

Figure S6 Effect of chemotherapies, anti-PD1 or anti-PDL1 Mabs and their combination on alternative immune checkpoints expression on CD8+ T cells in MBT-2, 4T1, MB49 and MC38 preclinical tumor models. Flow cytometric analysis of CD278 (ICOS) **(A)**, CD223 (LAG-3) **(B)**, CD279 (PD-1) **(C)**, CD274 (PDL-1) (**D**), TIGIT (**E**), and TIM-3 **(F)** on CD8+ T cells infiltrate of CD45+ cells. Mice were treated as in Table S1. Data are shown as mean values+ SEM, *n* = 5 to 6 mice/group **(A)**, *n* = 5 to 6 mice/group **(B)**, *n* = 6 mice/group **(C)**, *n* = 5 mice/group **(D)**.

Figure S7 Effect of chemotherapies, anti-PD1 or anti-PDL1 Mabs and their combination on alternative immune checkpoints expression on CD4+ T cells in MBT-2, 4T1, MB49, and MC38 preclinical tumor models. Flow cytometric analysis of CD278 (ICOS) **(A)**, CD223 (LAG-3) **(B)**, CD279 (PD-1) **(C)**, CD274 (PDL-1) **(D)**, TIGIT **(E)** and TIM-3 **(F)** on CD4+ T cells infiltrate of CD45+ cells. Mice were treated as in Table S1. Data are shown as mean values+ SEM, *n* = 5 to 6 mice/group **(A)**, *n* = 5 to 6 mice/group **(B)**, *n* = 6 mice/group **(C)**, *n* = 5 mice/group **(D)**. ^*^*p* < 0.05, ^**^*p* < 0.01, and ^***^*p* < 0.001 using Mann-Whitney test.

Figure S8 Effect of chemotherapies, anti-PD1 or anti-PDL1 Mabs and their combination on alternative immune checkpoints expression on tumor cells in MBT-2, 4T1, MB49 and MC38 preclinical tumor models. Flow cytometric analysis of CD278 (ICOS) **(A)**, CD223 (LAG-3) **(B)**, CD279 (PD-1) **(C)**, CD274 (PDL-1) **(D)**, TIGIT **(E)** and TIM-3 **(F)** on tumor cells infiltrate (CD45- cells). Mice were treated as in Table S1. Data are shown as mean values+ SEM, *n* = 5 to 6 mice/group **(A)**, *n* = 5 to 6 mice/group **(B)**, *n* = 6 mice/group **(C)**, *n* = 5 mice/group **(D)**. ^*^*p* < 0.05, ^**^*p* < 0.01, and ^***^*p* < 0.001 using Mann-Whitney test.

Table S1 *In vivo* experiments design overview.

Table S2 Flow cytometry assay design overview.

Table S3 Current Phase I-II clinical trials using anti-PD-1 or anti-PDL-1 in breast, colorectal and bladder cancer (from www.clinicaltrials.gov, searched items “nivolumab” and “atezolizumab”, 2018, ICI = Immune Checkpoint Inhibitor).
