# Supplementary figures and images for: The Antitumor Activity of Combinations of Cytotoxic Chemotherapy and Immune Checkpoint Inhibitors Is Model-Dependent
Source: Front Immunol. 2018 Oct 9;9:2100. doi: 10.3389/fimmu.2018.02100 (PMC6190749; doi:10.3389/fimmu.2018.02100)

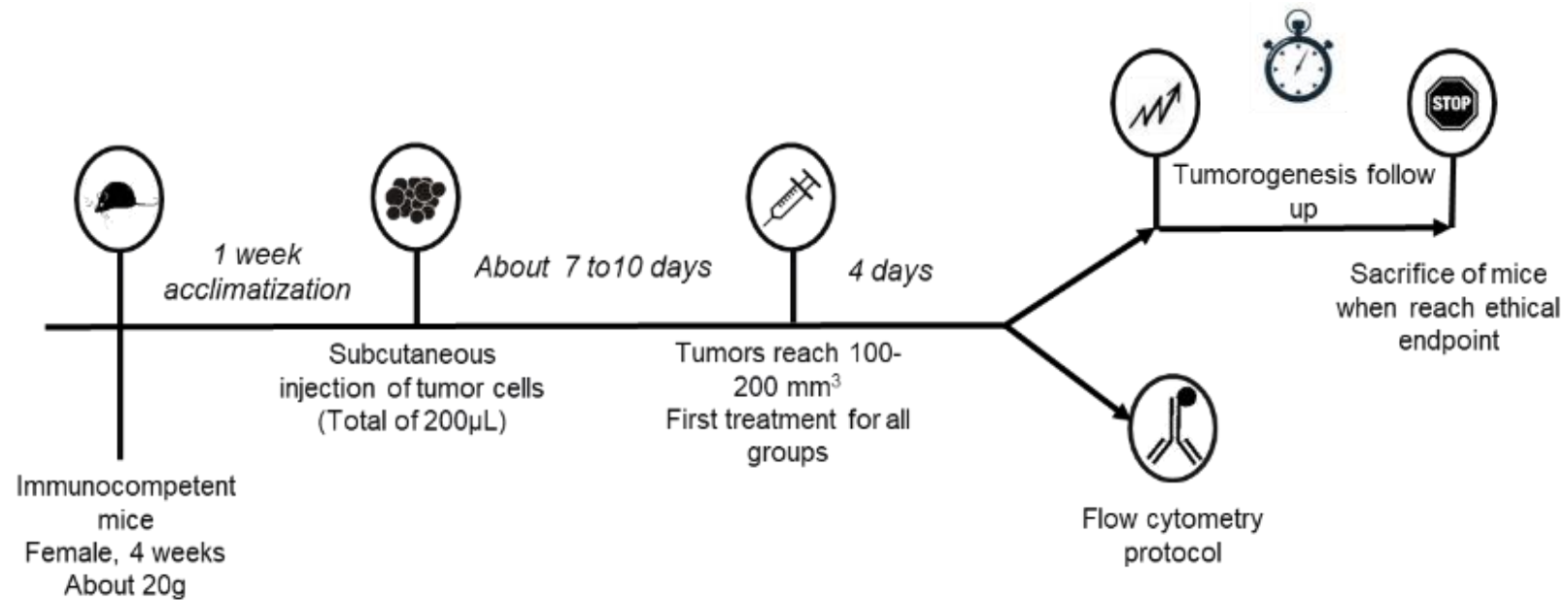

Supplement: Figure S1 — Experimental design of combination experiments. [file Image_1.PDF]

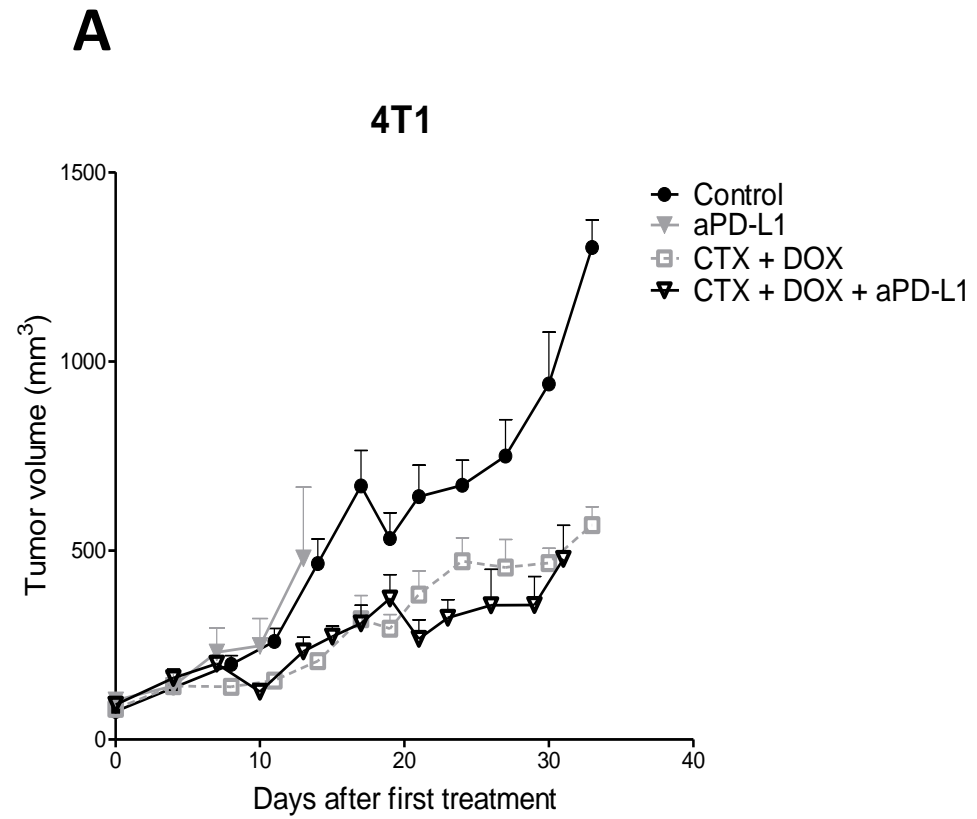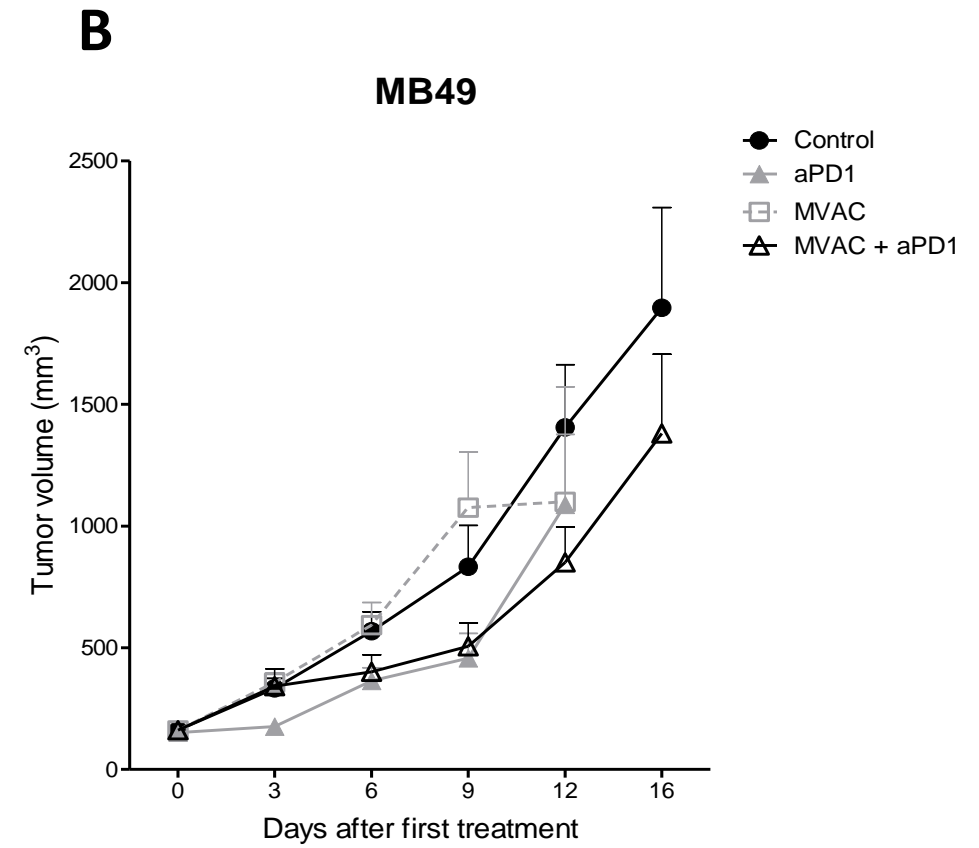

Supplement: Figure S2 — Effect of anti-PD1 (clone RMP1.14) or anti-PDL1 (clone 10F.9G2) antibody, 12,5 mg/kg, i.p., q1wk, in combination with cyclophosphamide (CTX) 100 mg/kg, i.p, q1wk and doxorubicin (DOX) 2 mg/kg, i.p, q1wk in SC metastatic breast cancer 4T1 (A), methotrexate 1mg/kg, i.p, q1wk and vinblastine 0,1 mg/kg, i.p, q1wk and doxorubicin 1 mg/kg, i.p, q1wk and cisplatin 1 mg/kg i.p, q1wk (MVAC) in SC bladder cancer MB49 (B), Data are shown as Mean + SEM, n = 5 to 6 (A), n = 6 (B). [file Image_2.PDF]

Tube 1

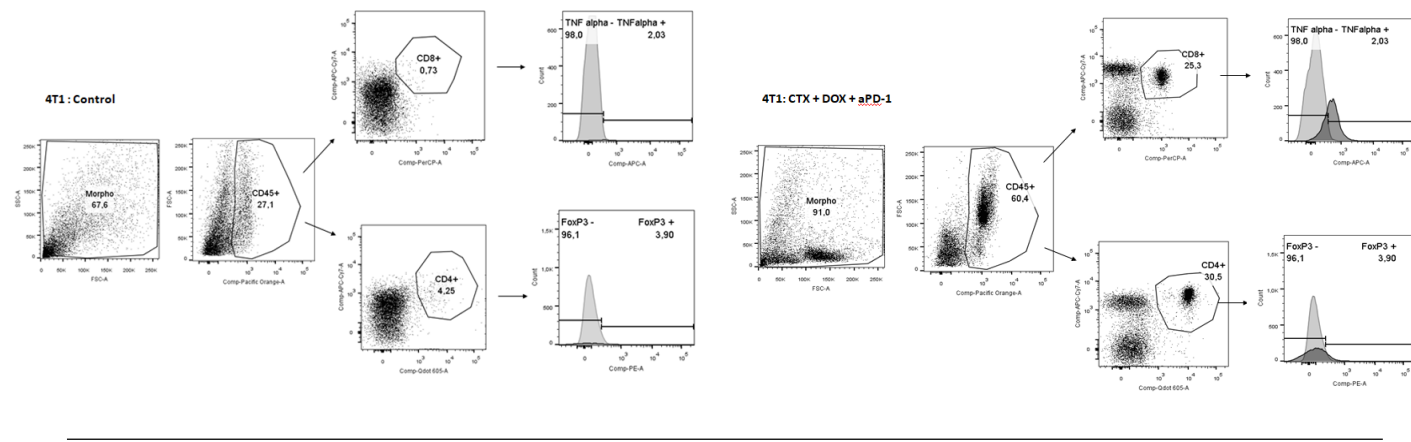

Tube 2

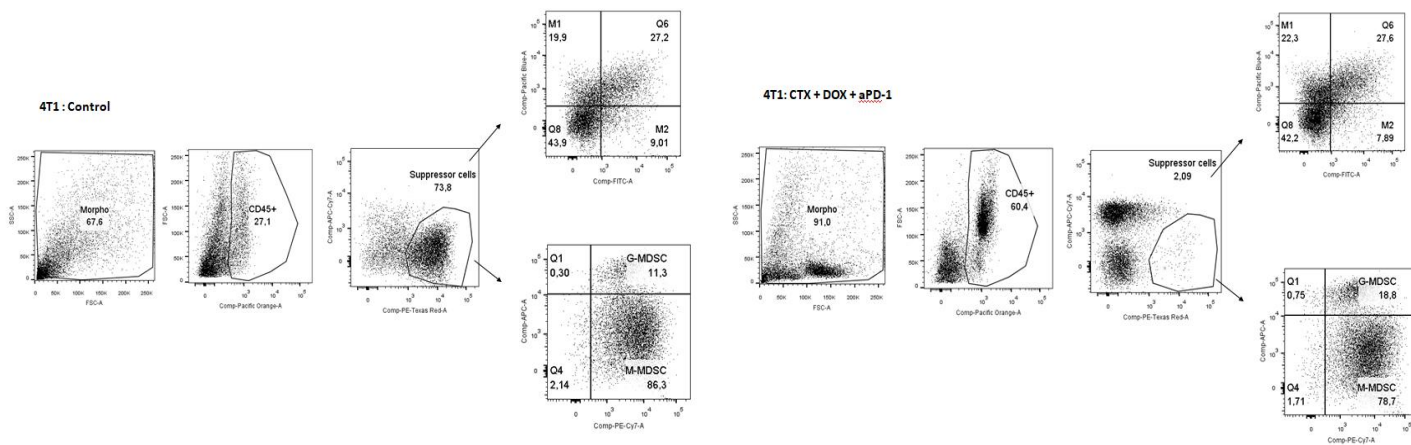

Tube 3

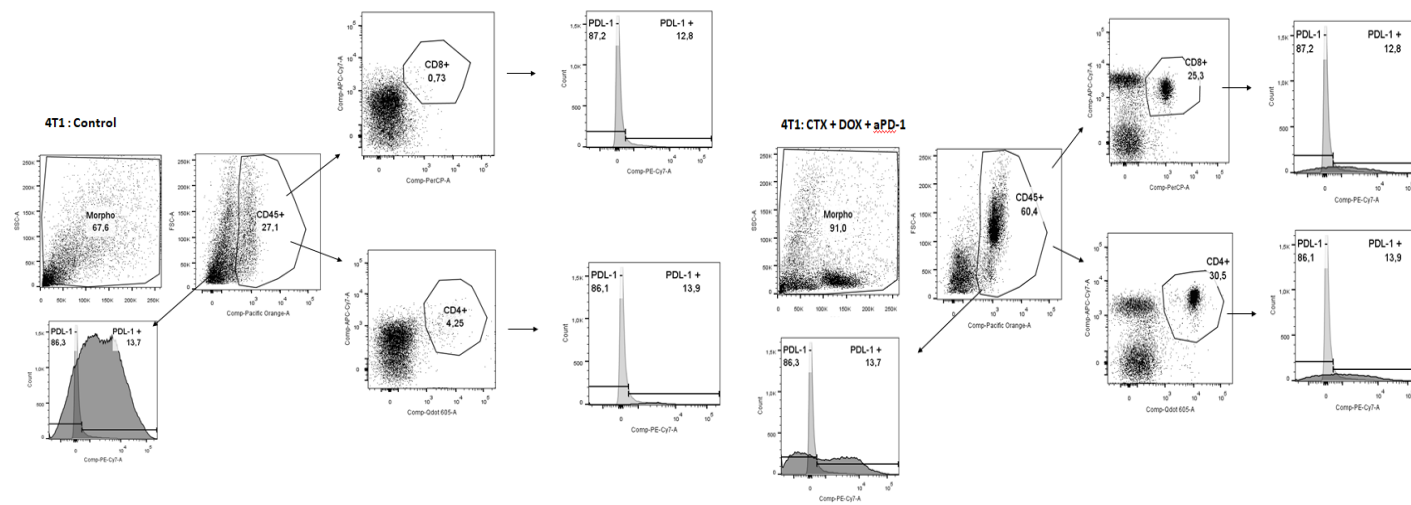

Supplement: Figure S3 — Example of gating strategy for immune cell infiltrate study. Example of flow cytometry analysis results: comparison between Control tumor and tumor treated with cyclophosphamide 100 mg/kg i.p and doxorubicin 2 mg/kg i.p and anti-PD1 12.5 mg/kg i.p. (CTX + DOX + PD1) in SC breast cancer 4T1. [file Image_3.PDF]

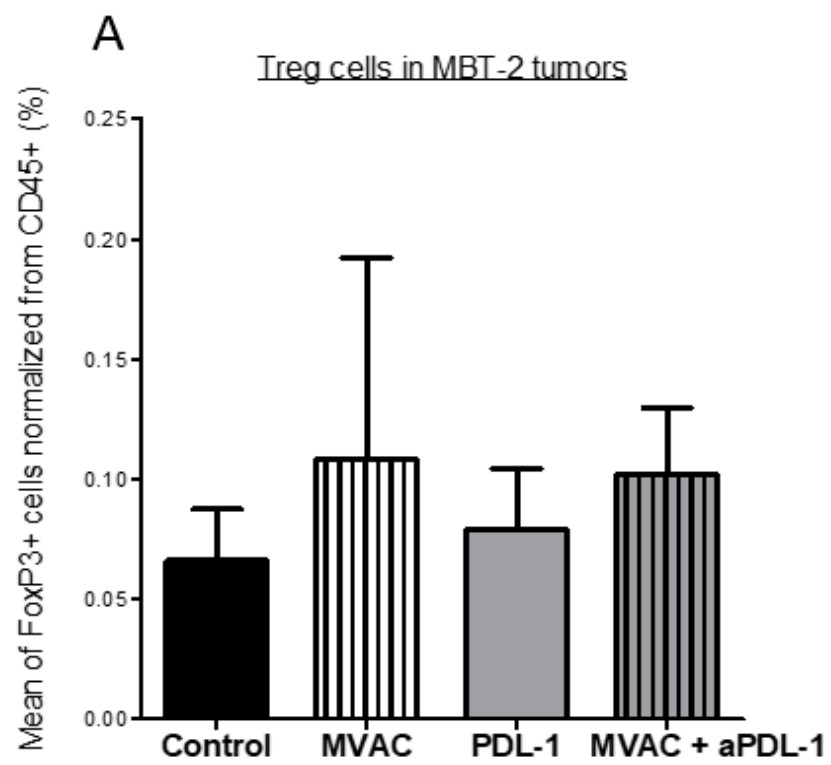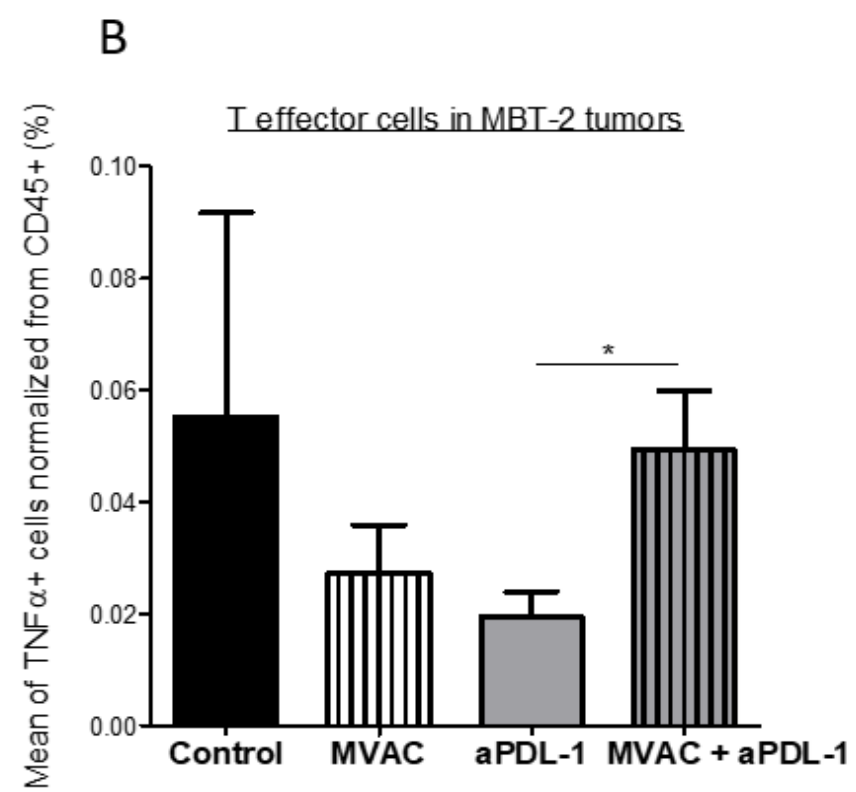

Supplement: Figure S4 — Effect of chemotherapies, anti-PDL1 Mab and their combination on T reg cells (A) and T effector cells activation (B) in MBT-2 preclinical tumor model. Flow cytometric analysis of TNFα+ CD8+ T cells of total CD45+ cells (A), FoxP3+ CD4+ T cells of total CD45+ cells (B). Mice were treated as in Table S1. Data are shown as Mean + SEM, n = 5 to 6, Mann–Whitney test: *P < 0.05. [file Image_4.PDF]

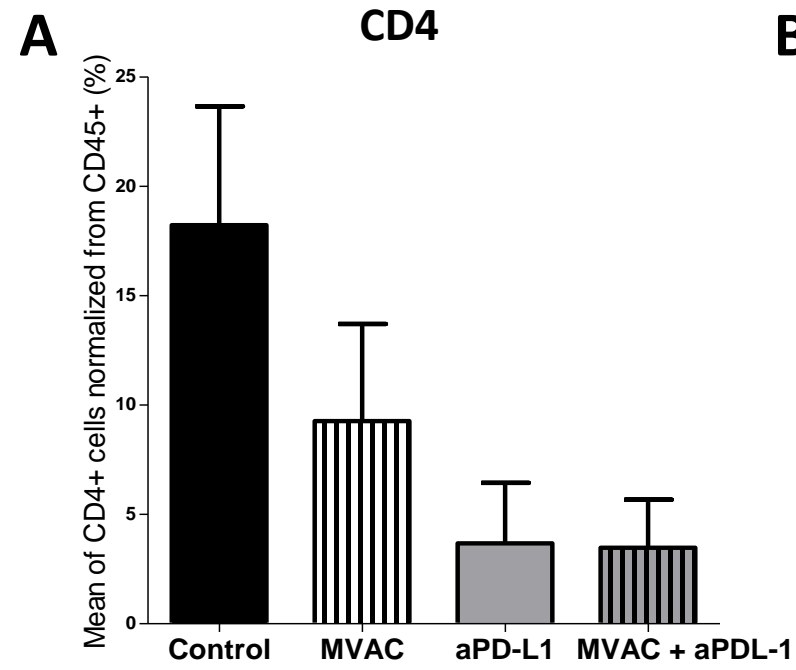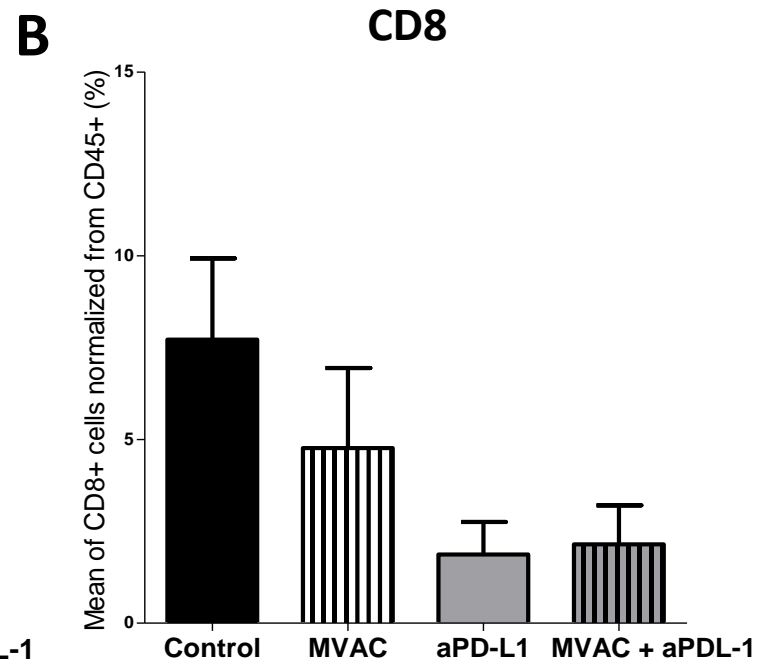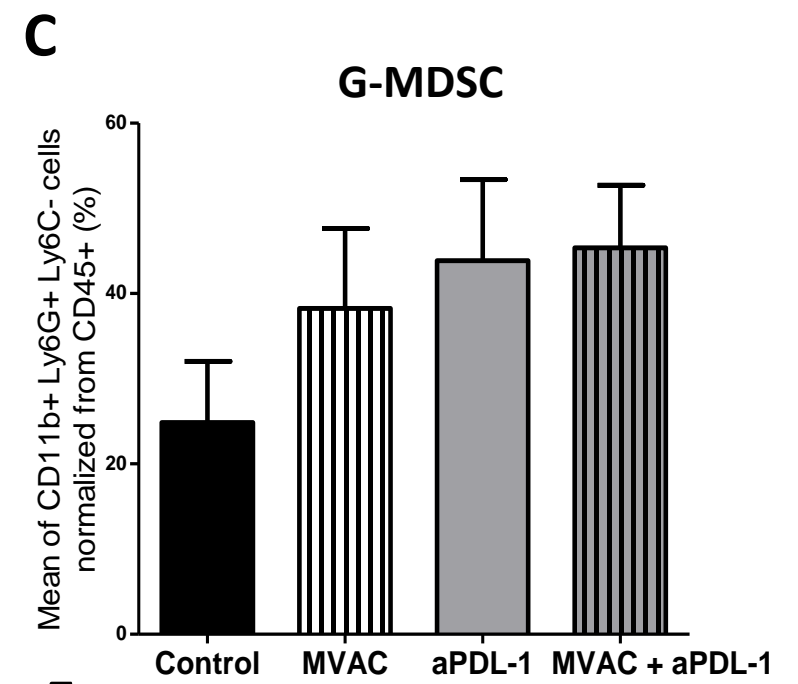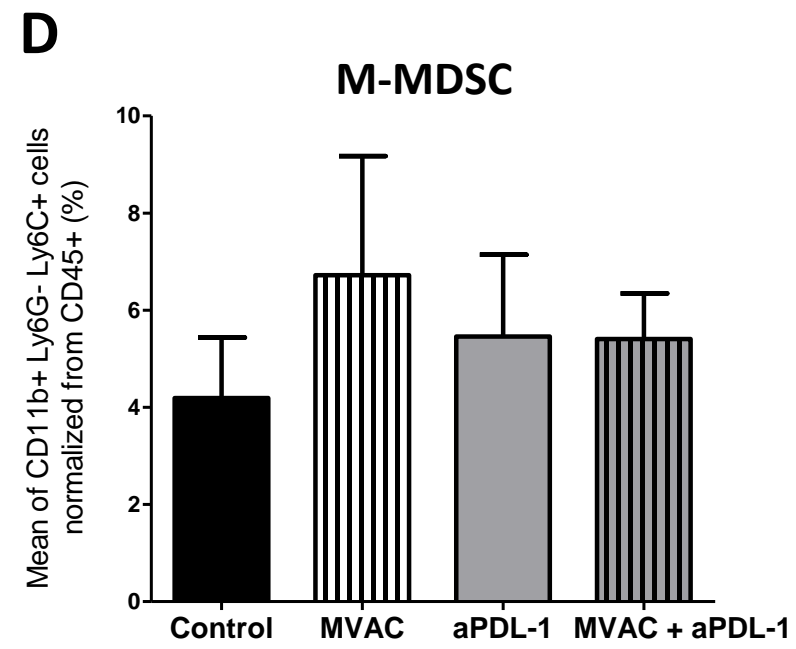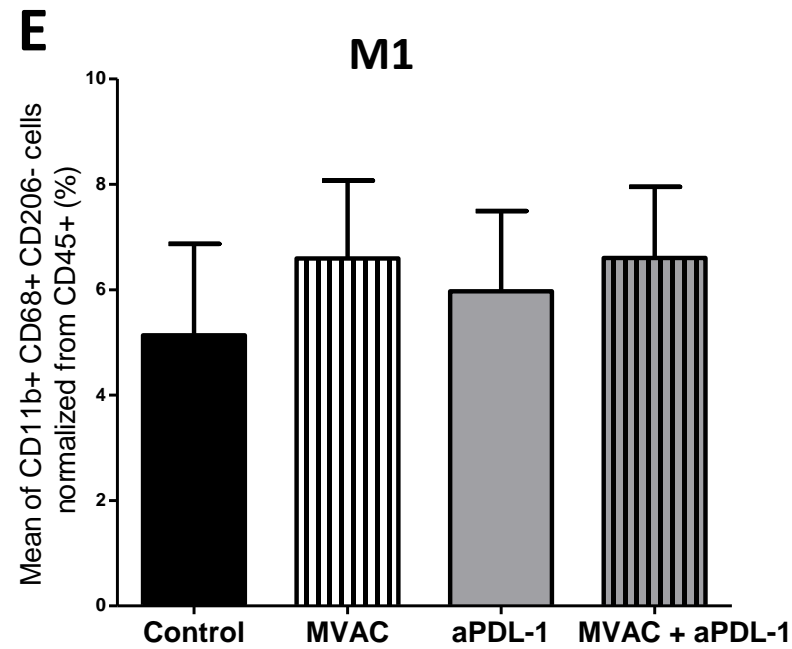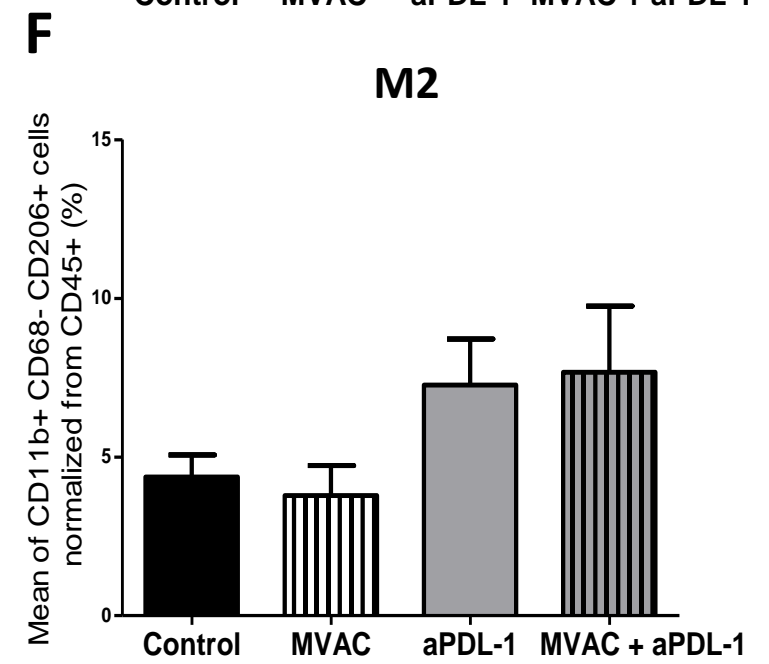

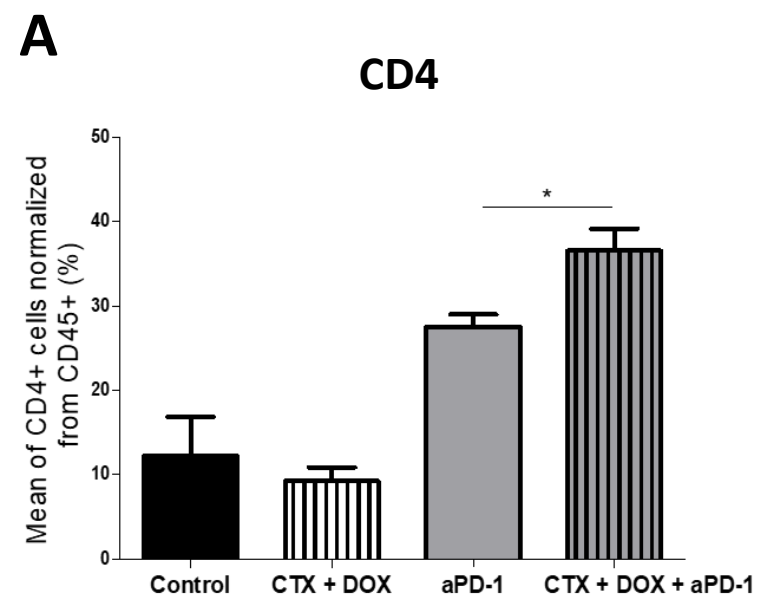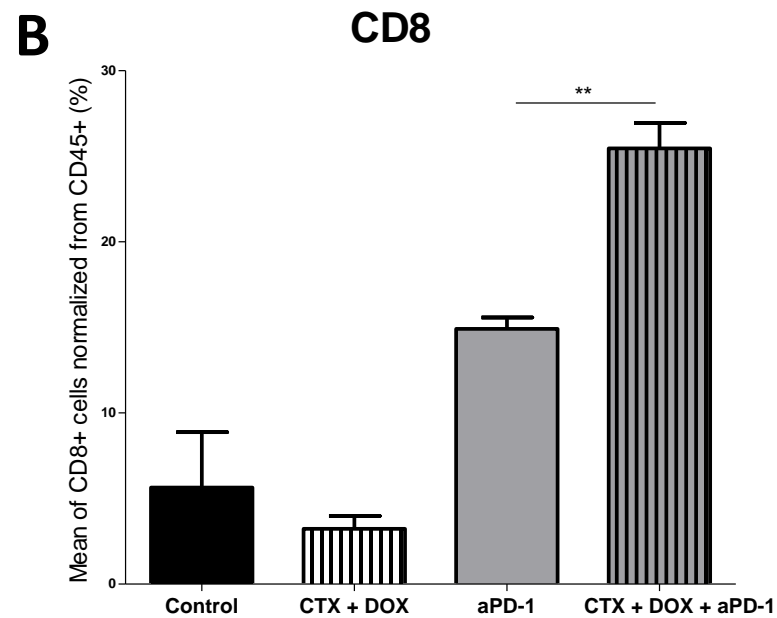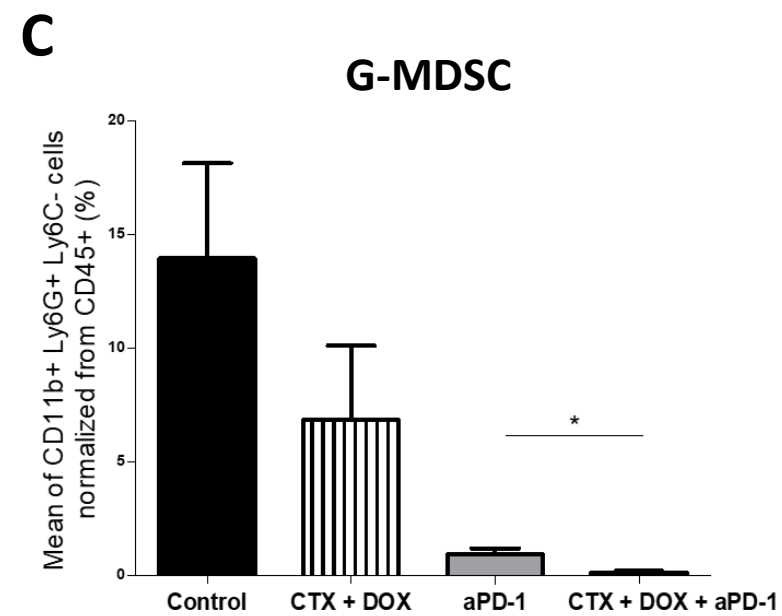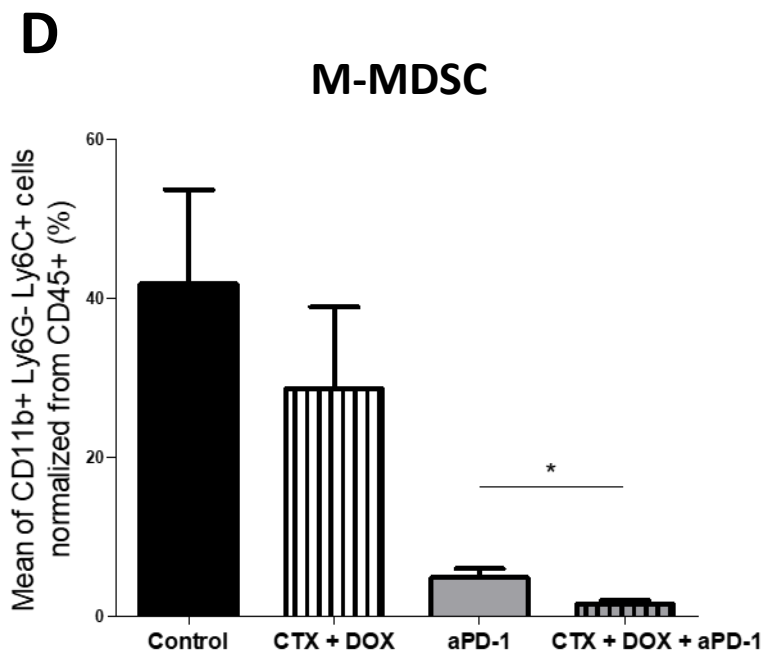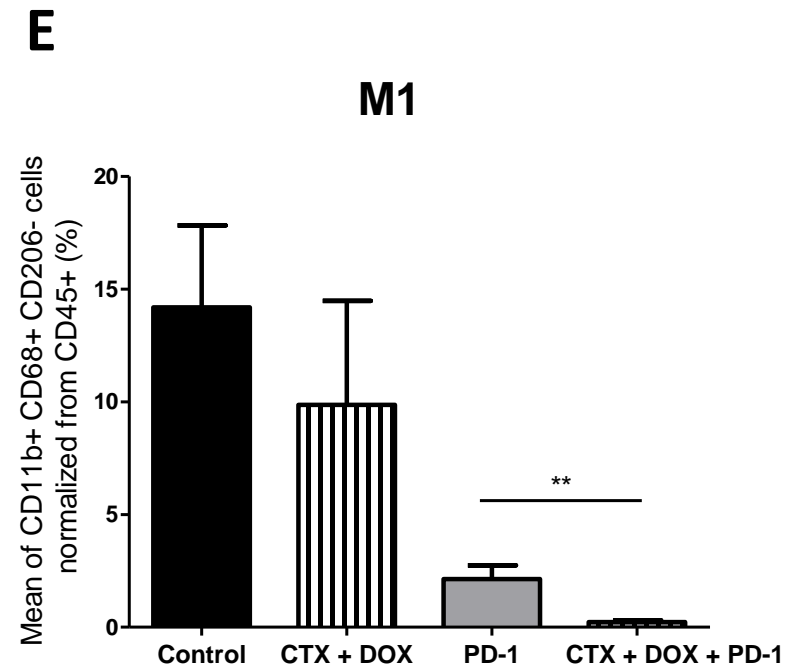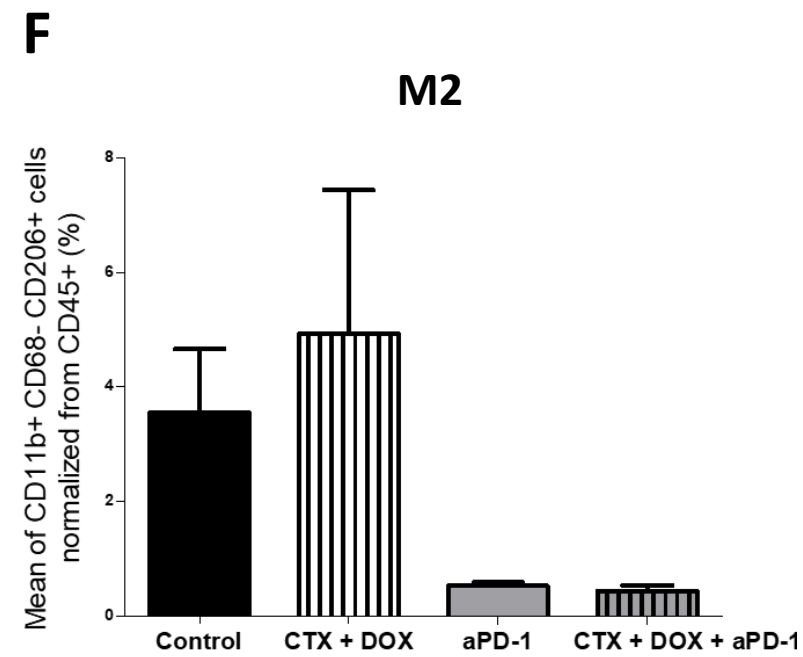

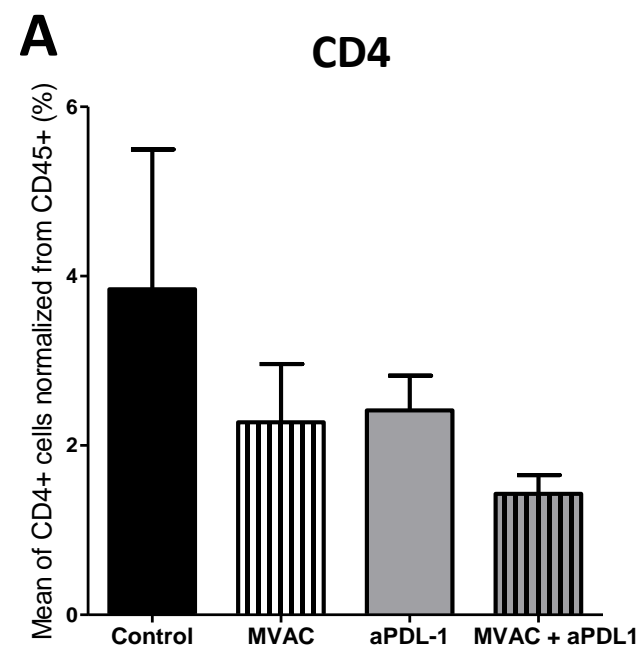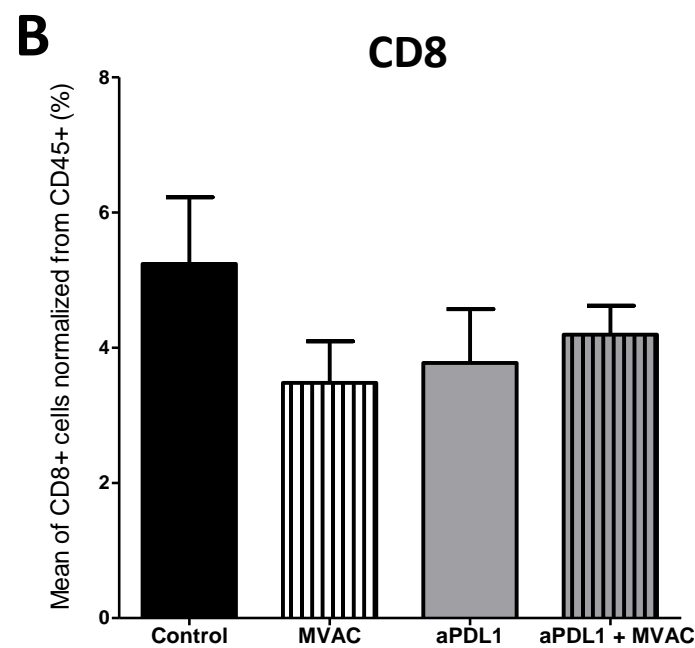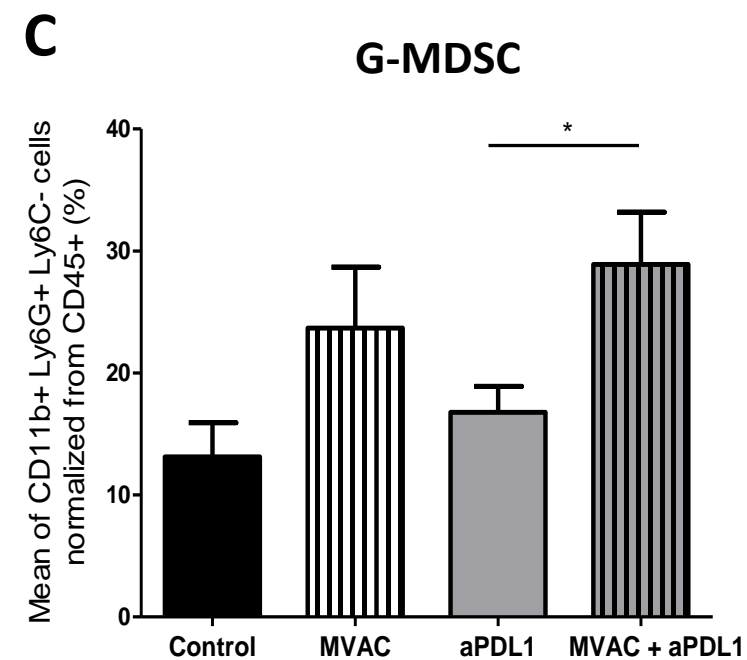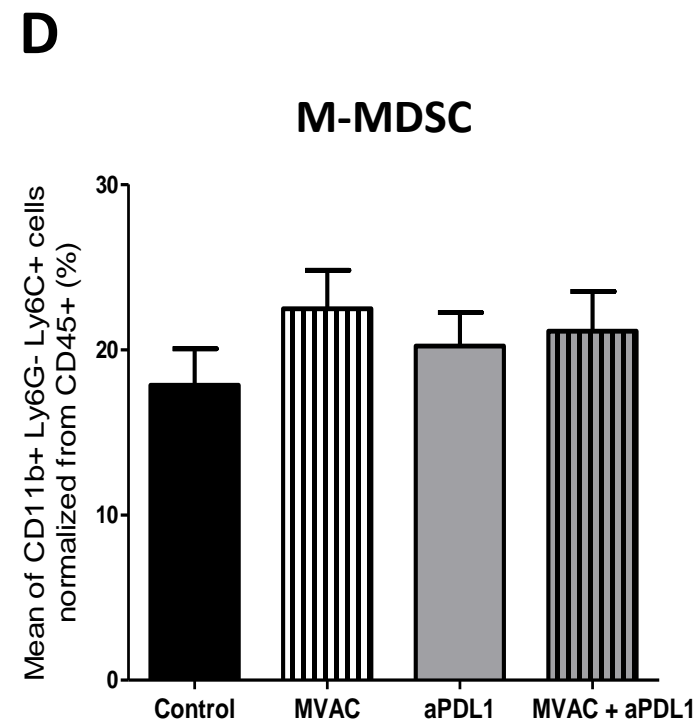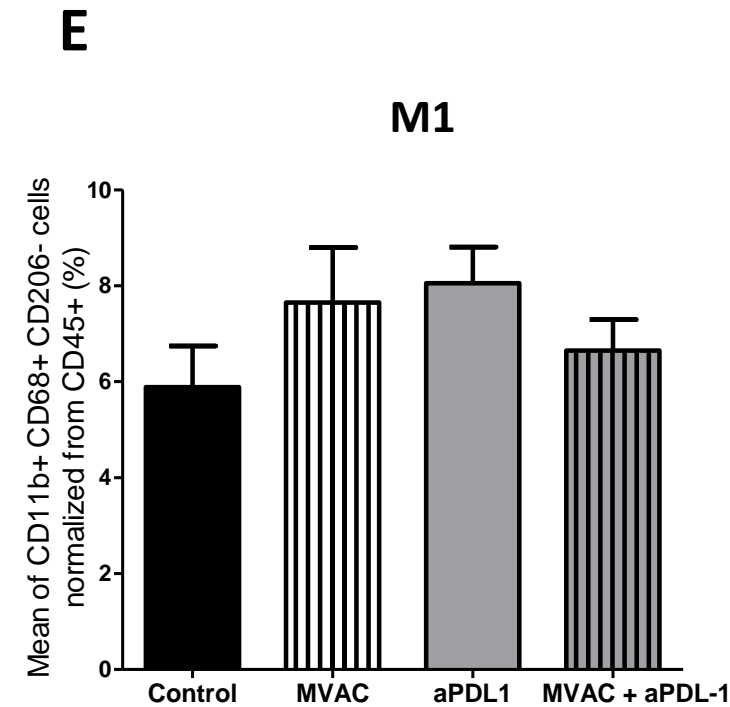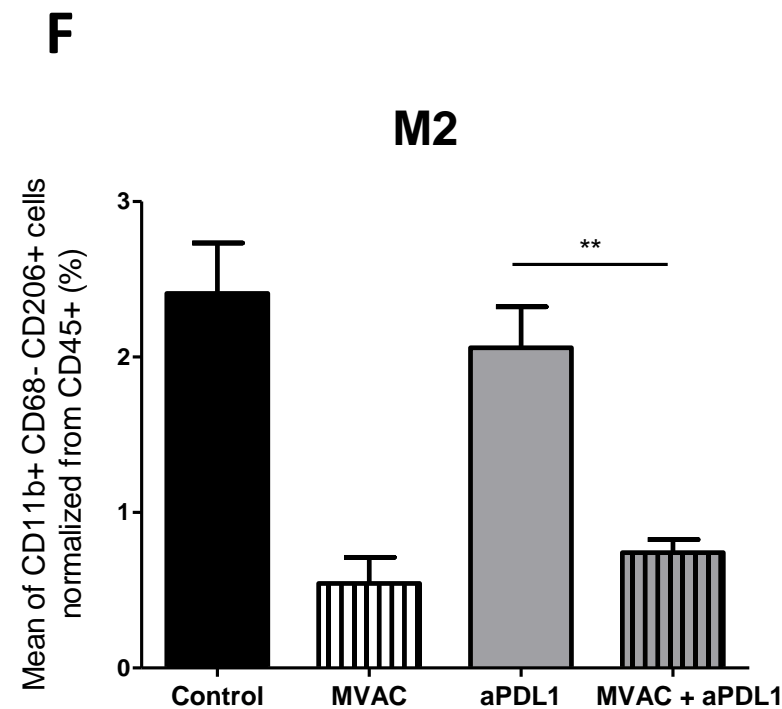

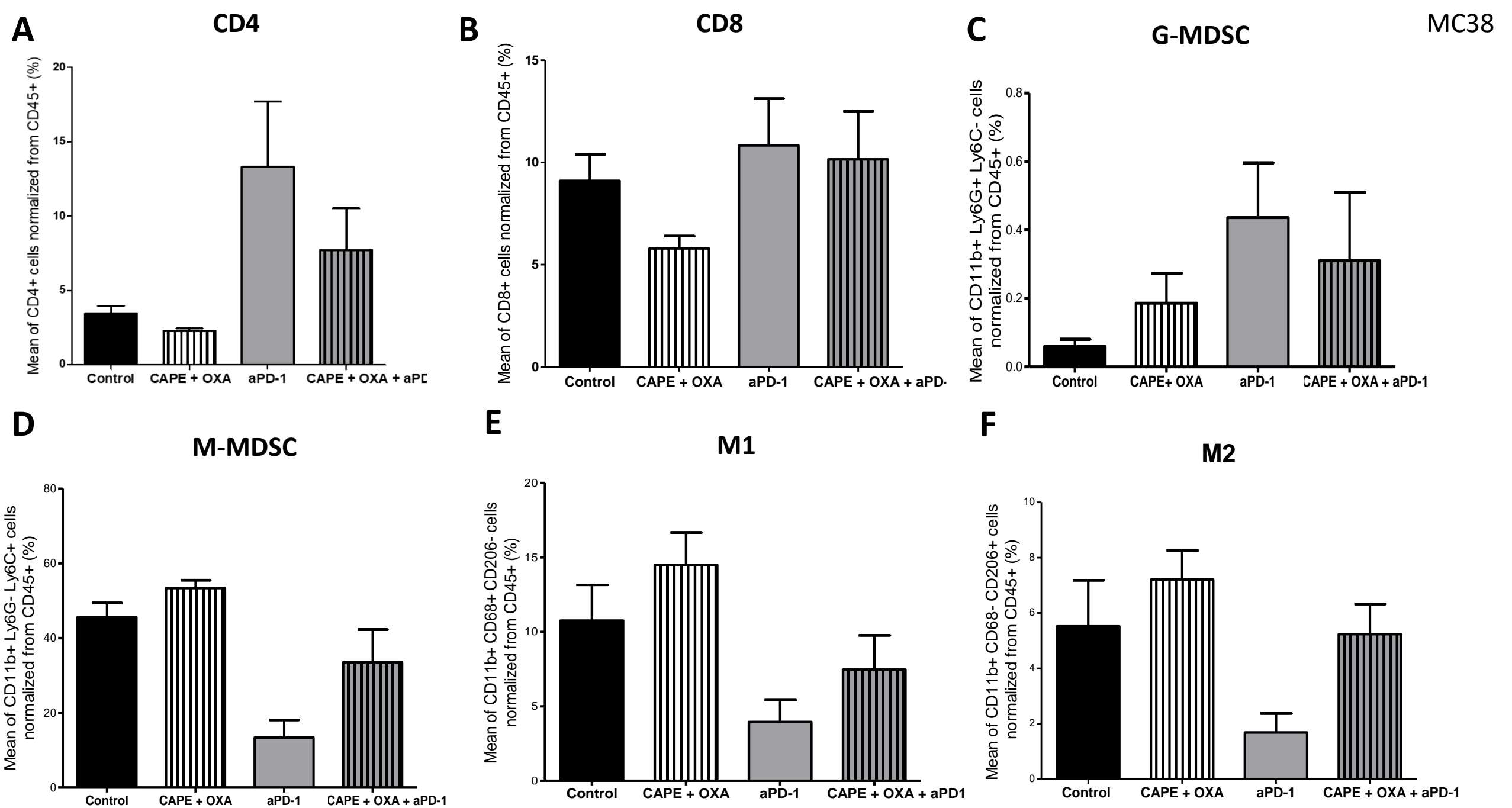

Supplement: Figure S5 — Effect of chemotherapies, anti-PD1 or anti-PDL1 mAbs and their combination on leucocyte infiltrate subpopulations in MBT-2, 4T1, MB49, and MC38 preclinical tumor models. Flow cytometric analysis of total CD4+ T cells: CD45+CD3+CD4+ (A), CD8+ T cells CD45+ CD3+CD8+ (B),Granulocytic Myeloid Derived Suppressor Cells (G-MDSC): CD45+ CD3- CD11b+ Ly6G+ Ly6C- (C) Monocytic Myeloid Derived Suppressor Cells (M-MDSC): CD45+ CD3- CD11b+ Ly6G- Ly6C+ (D), M1 macrophages: CD45+ CD3- CD11b+ CD68+ CD206- (E)), M2 macrophages: CD45+ CD3- CD11b+ CD68- CD206+ (F). Mice were treated as in Table S1. Data are shown as mean values+ SEM, n = 5 to 6 mice/group (A), n = 5 to 6 mice/group (B), n = 6 mice/group (C), n = 5 mice/group (D). *p < 0.05 and **p < 0.01 using Mann-Whitney test. [file Image_5.PDF]

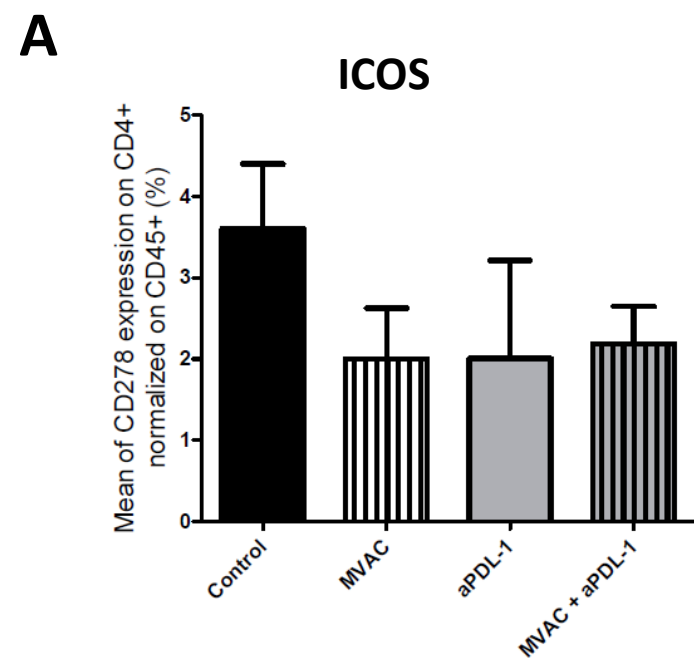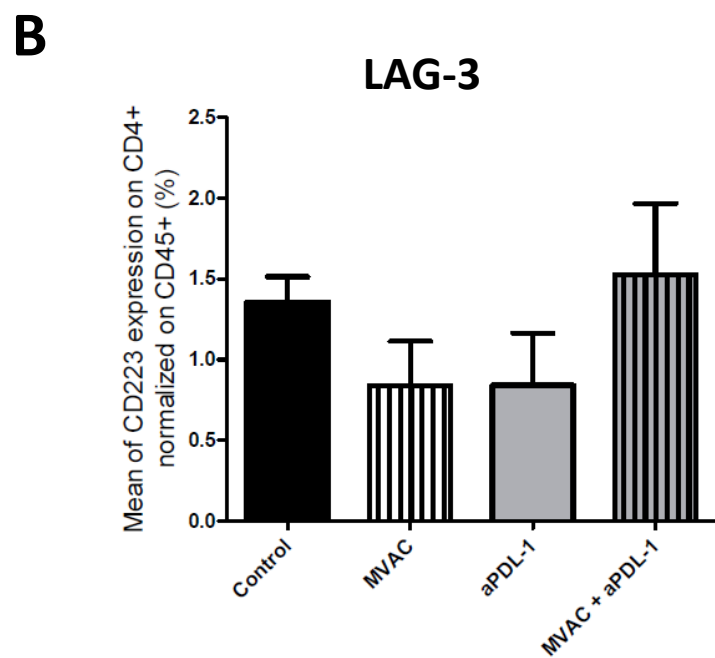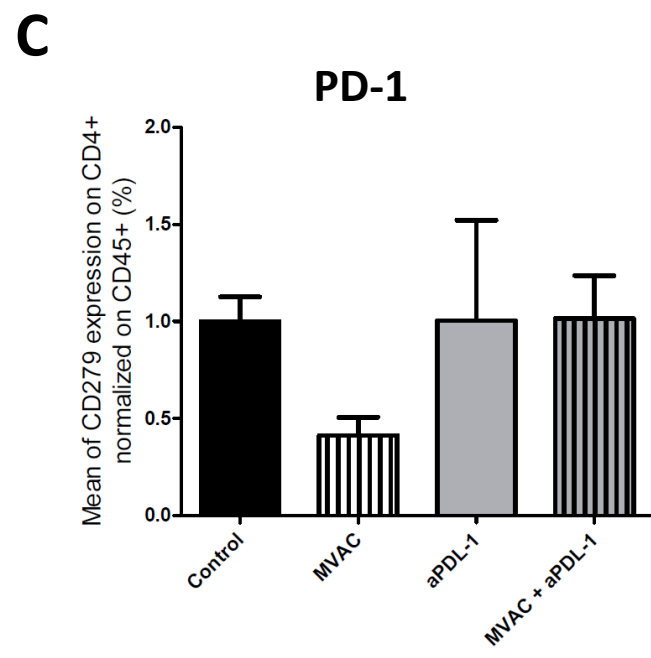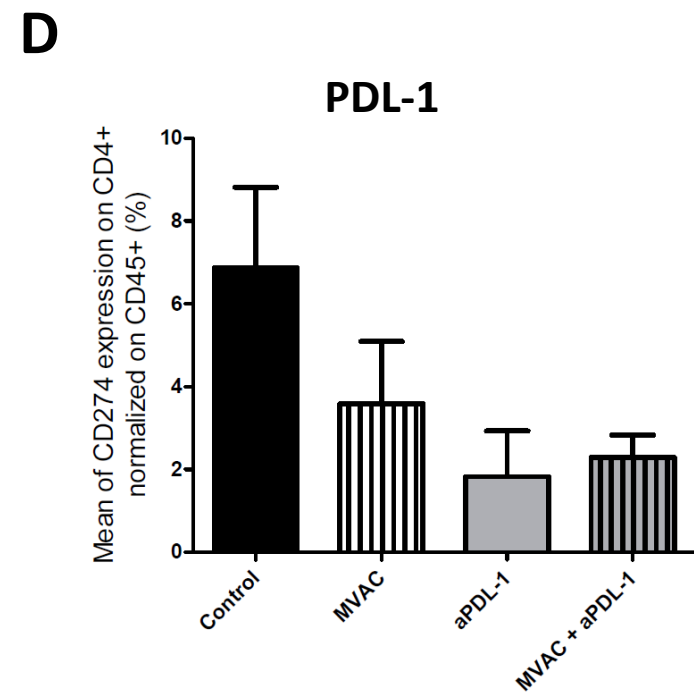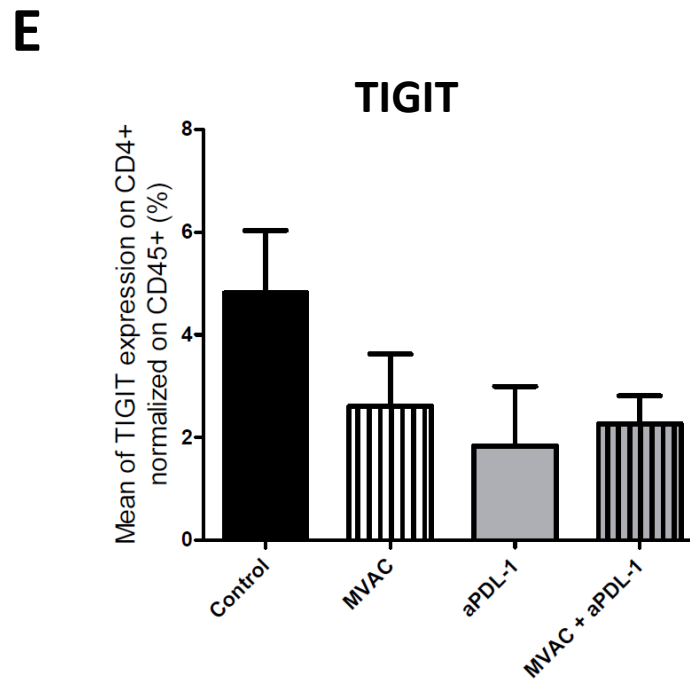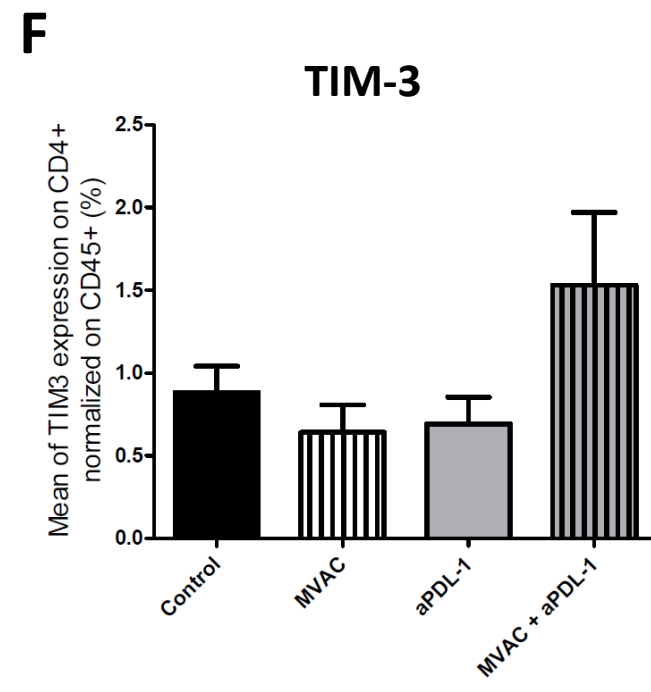

**A**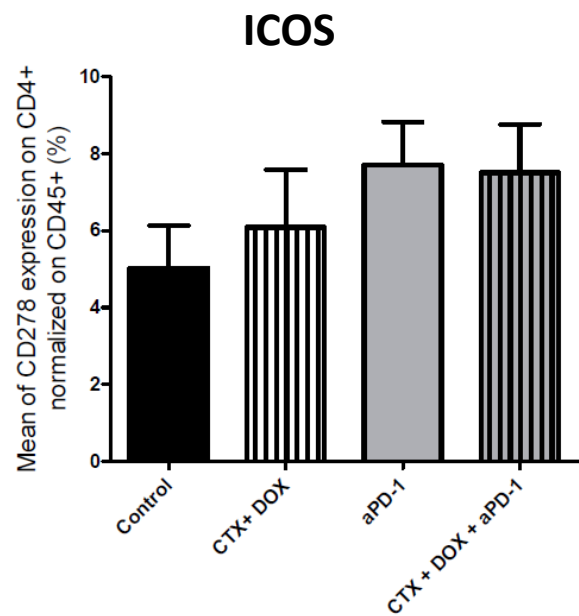**B**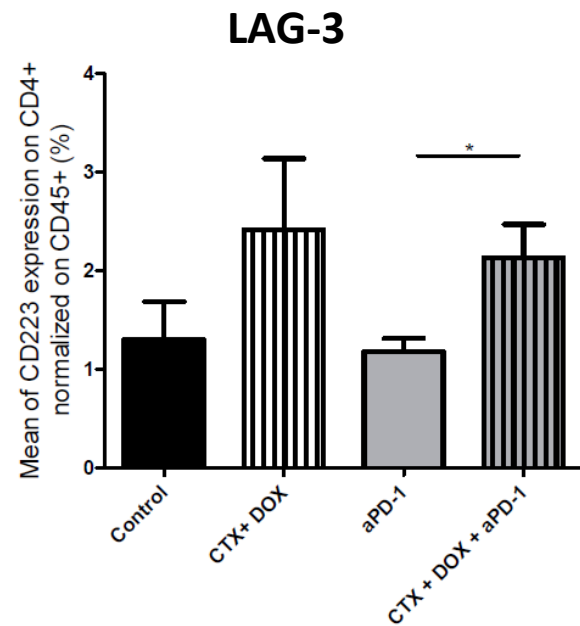**C**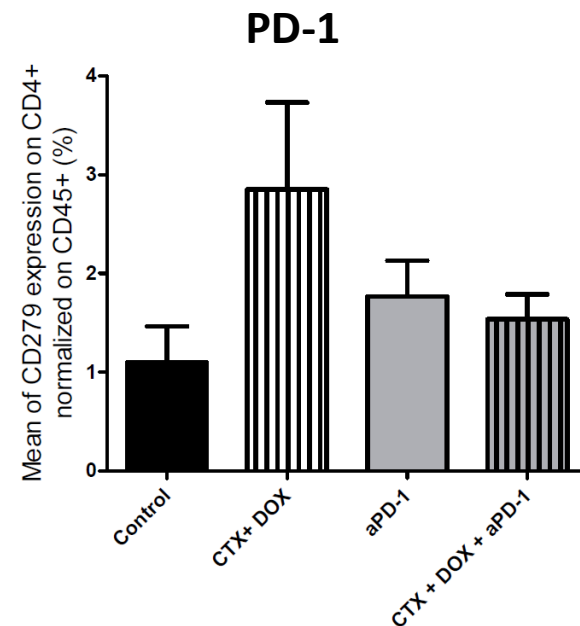**D**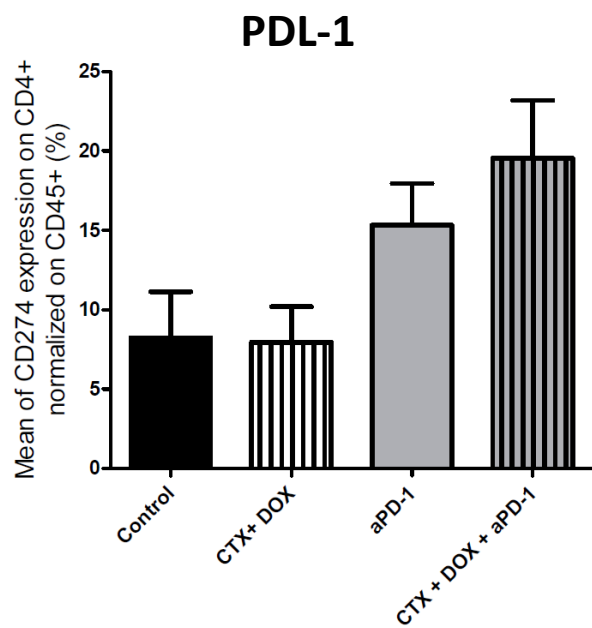**E**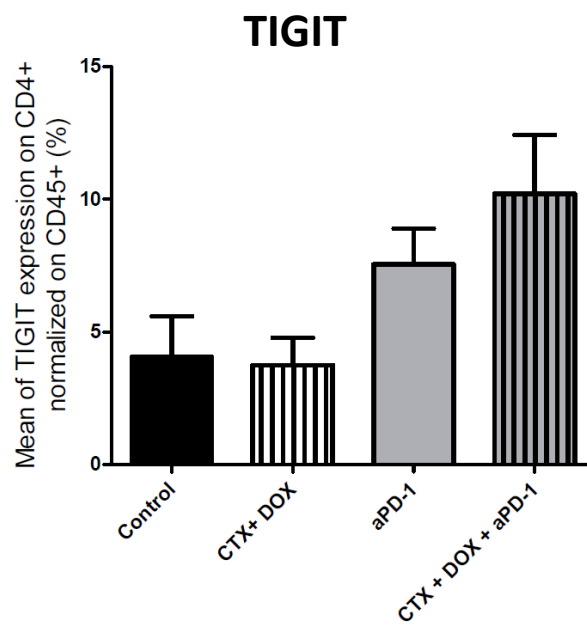**F**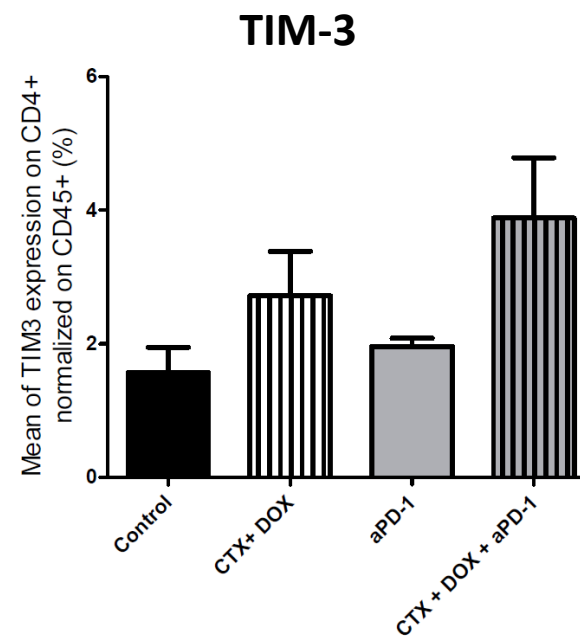

**A**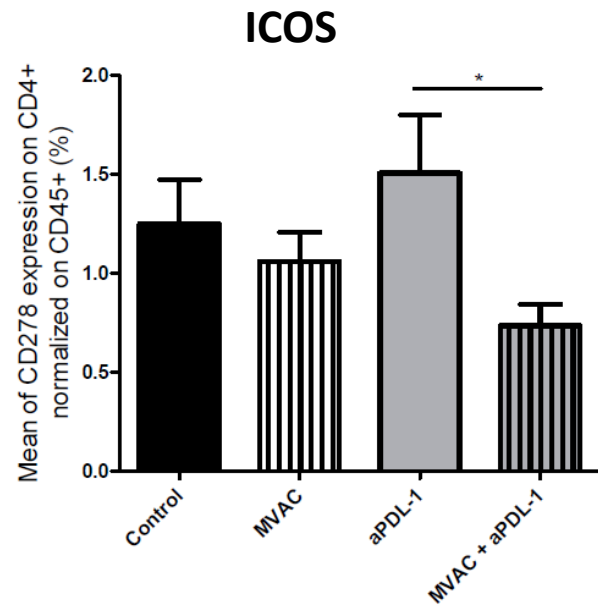**B**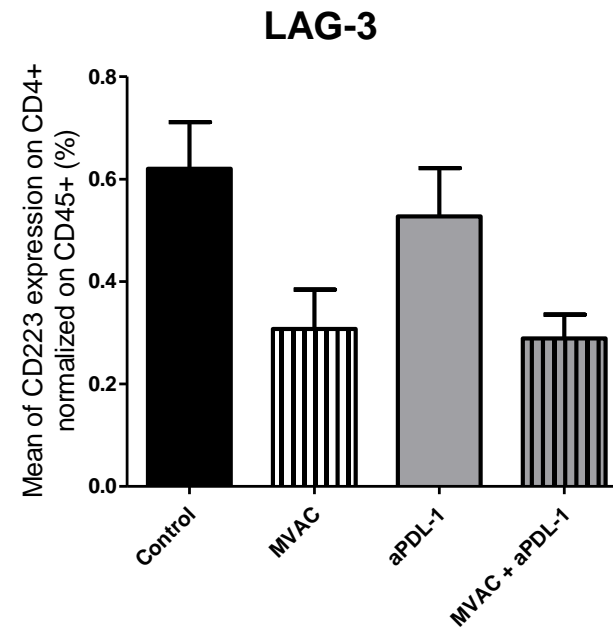**C**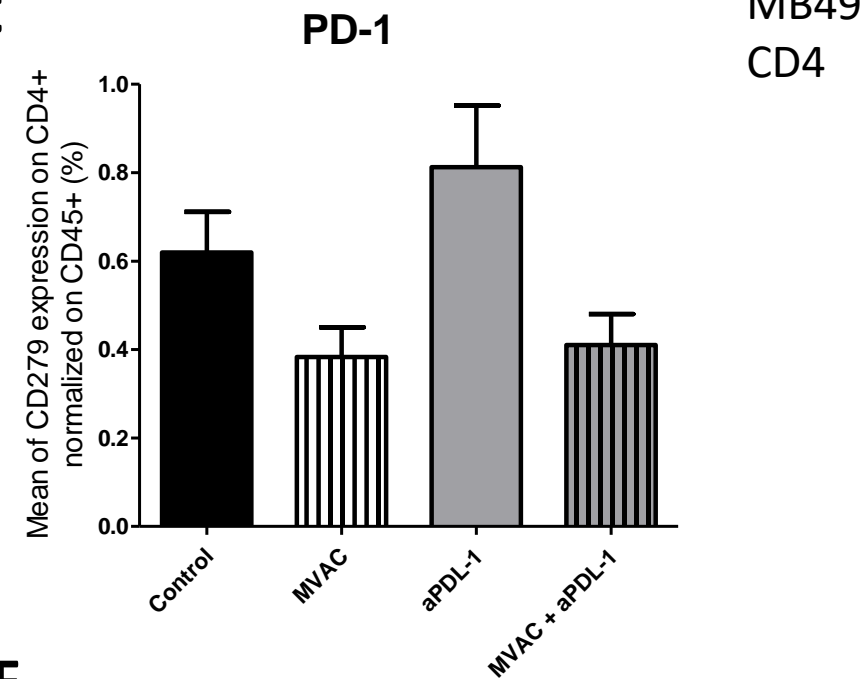**D**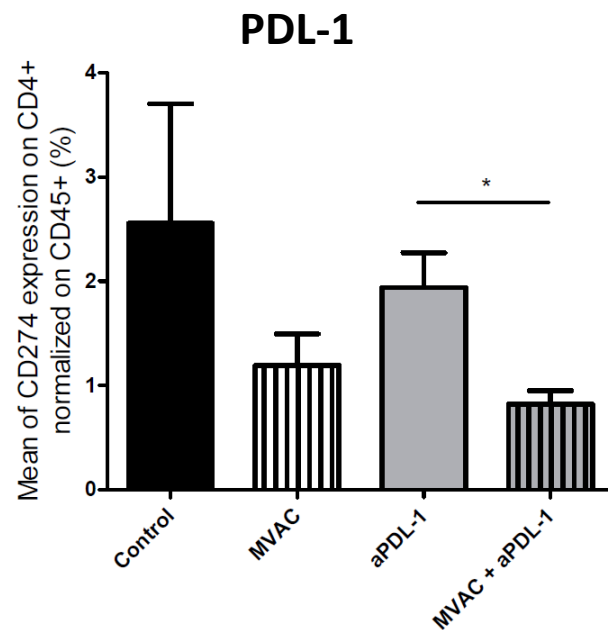**E**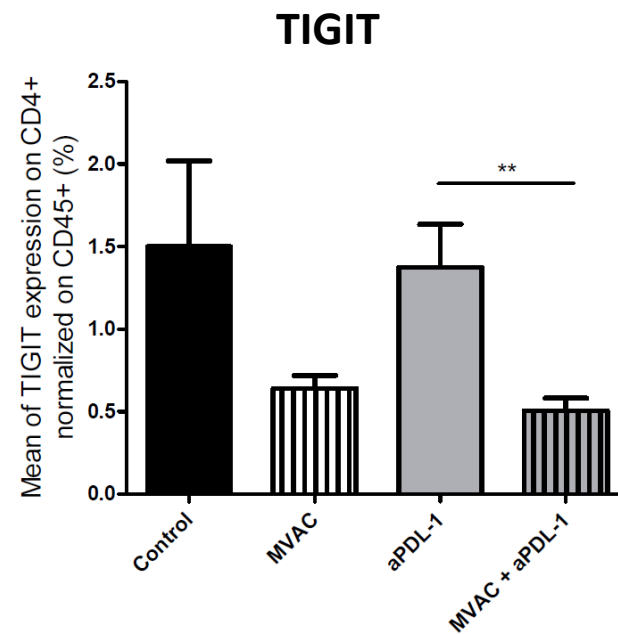**F**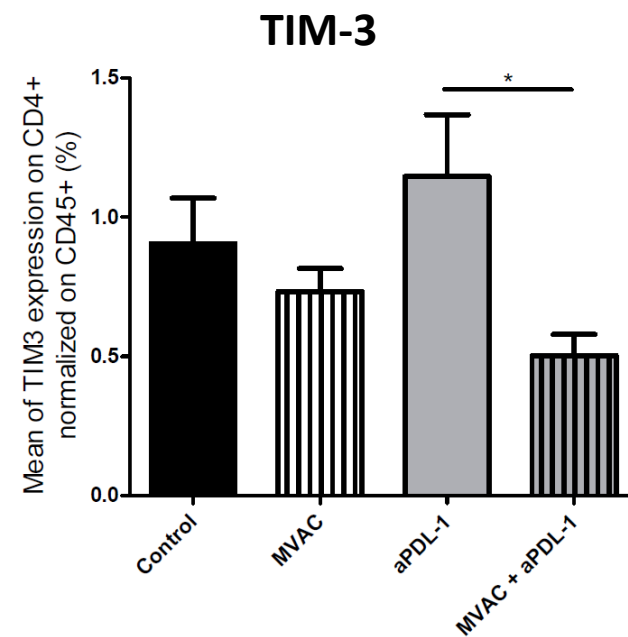

**A****ICOS**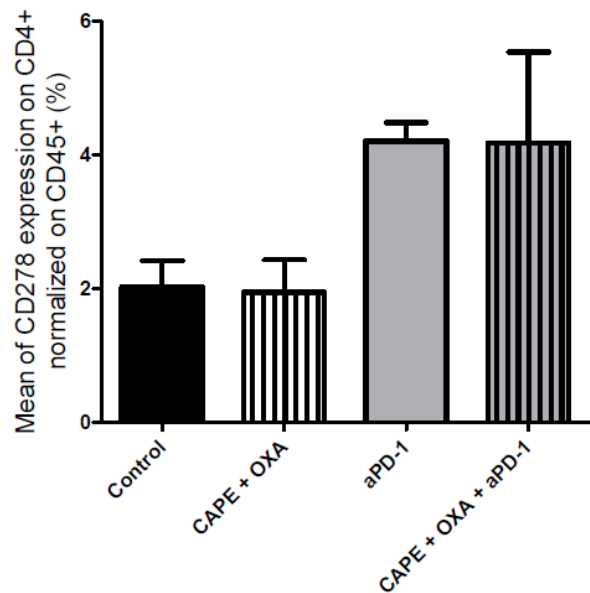**B****LAG-3**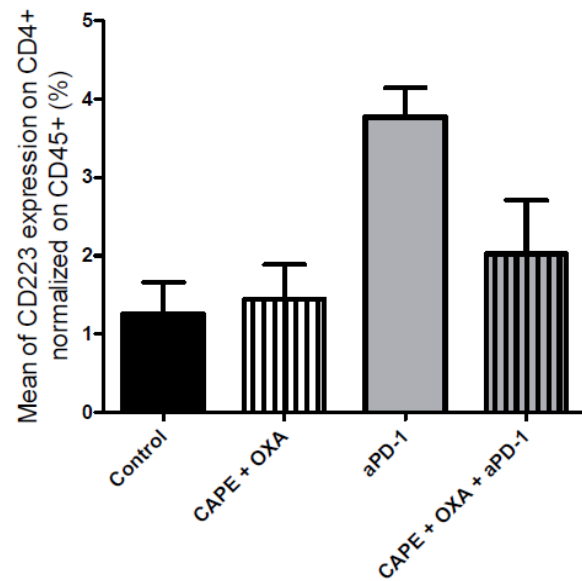**C****PD-1**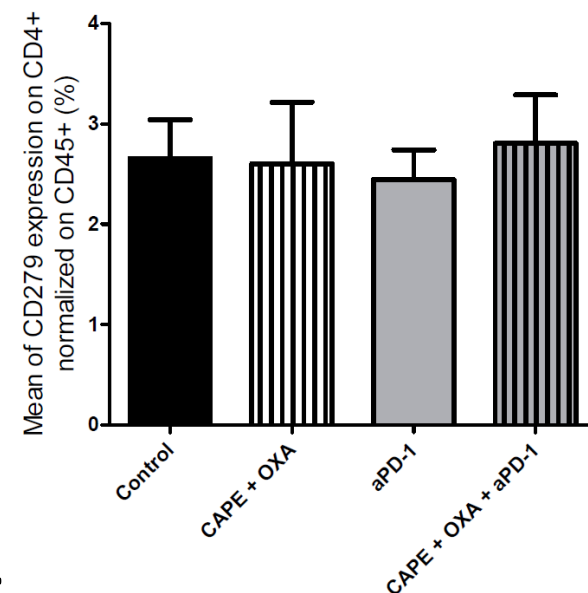**D****PDL-1**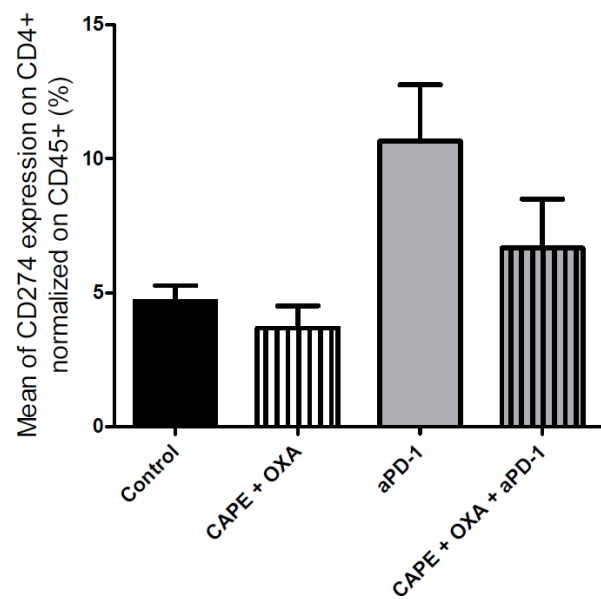**E****TIGIT**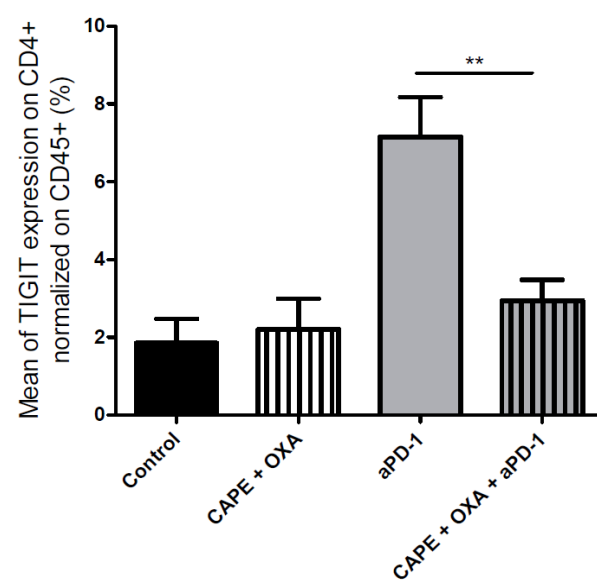**F****TIM-3**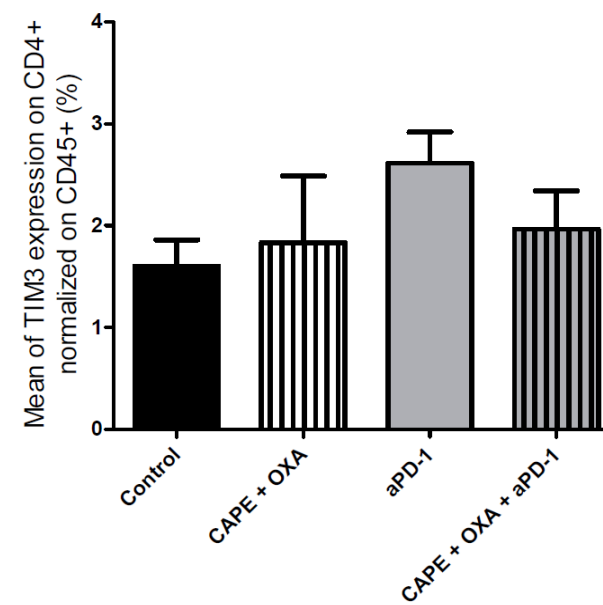

Supplement: Figure S7 — Effect of chemotherapies, anti-PD1 or anti-PDL1 Mabs and their combination on alternative immune checkpoints expression on CD4+ T cells in MBT-2, 4T1, MB49, and MC38 preclinical tumor models. Flow cytometric analysis of CD278 (ICOS) (A), CD223 (LAG-3) (B), CD279 (PD-1) (C), CD274 (PDL-1) (D), TIGIT (E) and TIM-3 (F) on CD4+ T cells infiltrate of CD45+ cells. Mice were treated as in Table S1. Data are shown as mean values+ SEM, n = 5 to 6 mice/group (A), n = 5 to 6 mice/group (B), n = 6 mice/group (C), n = 5 mice/group (D). *p < 0.05, **p < 0.01, and ***p < 0.001 using Mann-Whitney test. [file Image_7.PDF]

**A**

**ICOS**

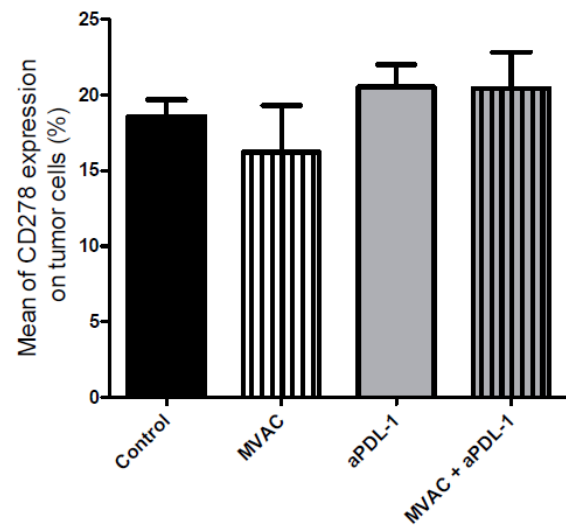

**B**

**LAG-3**

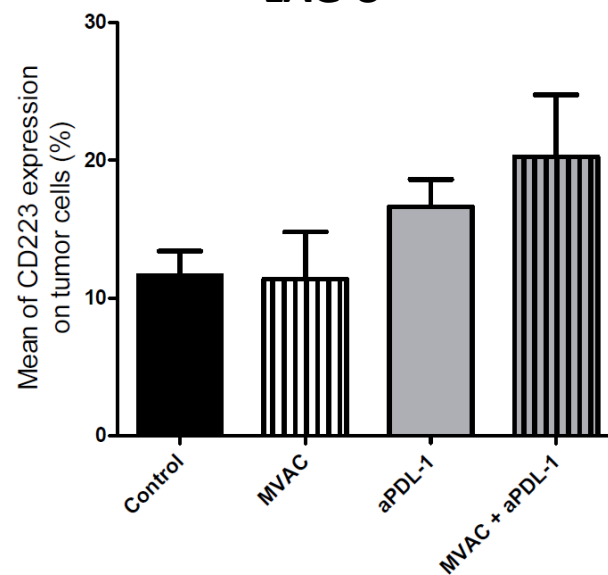

**C**

**PD-1**

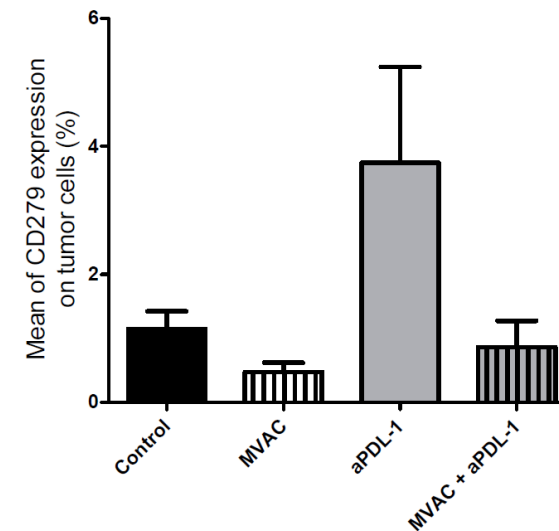

**D**

**PDL-1**

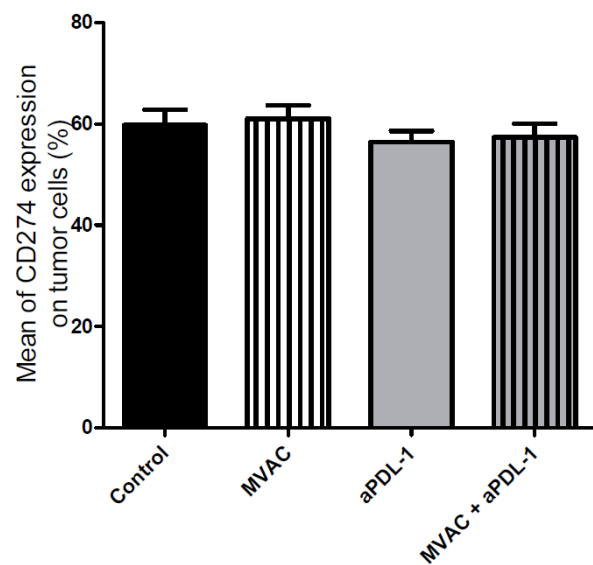

**E**

**TIGIT**

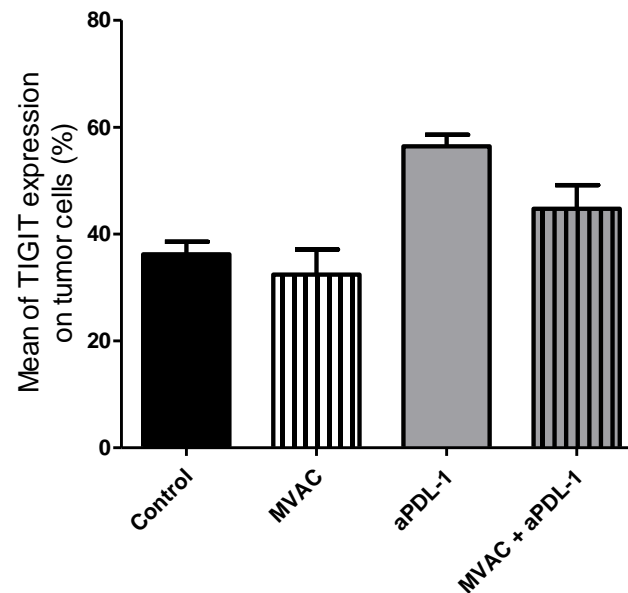

**F**

**TIM-3**

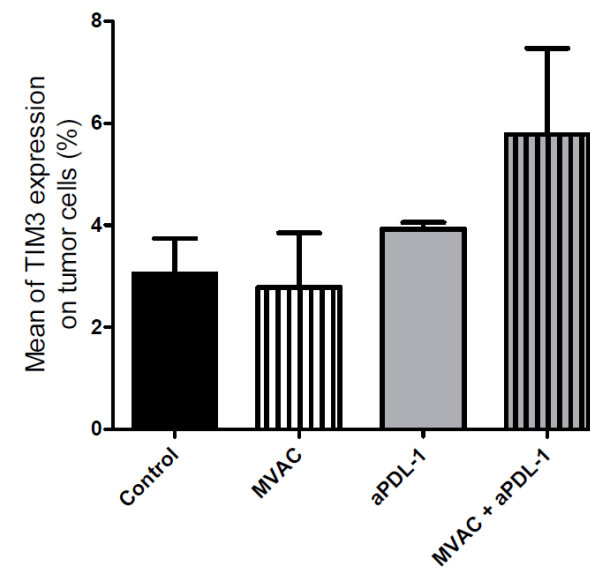

**A**

**ICOS**

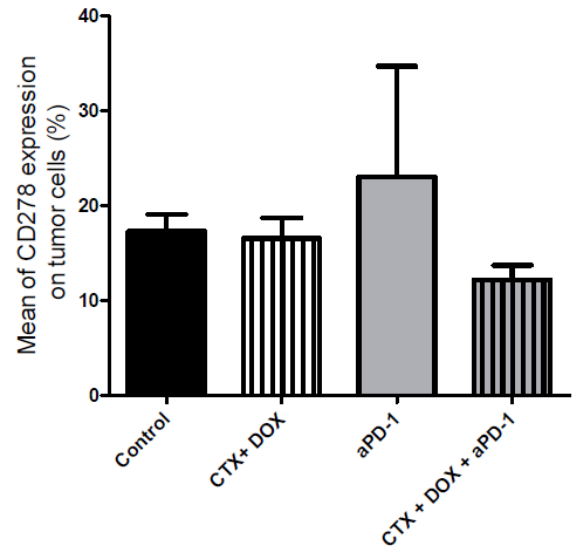

**B**

**LAG-3**

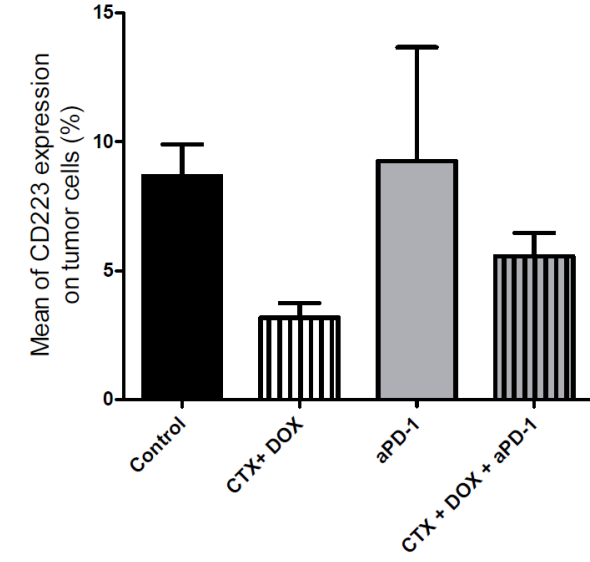

**C**

**PD-1**

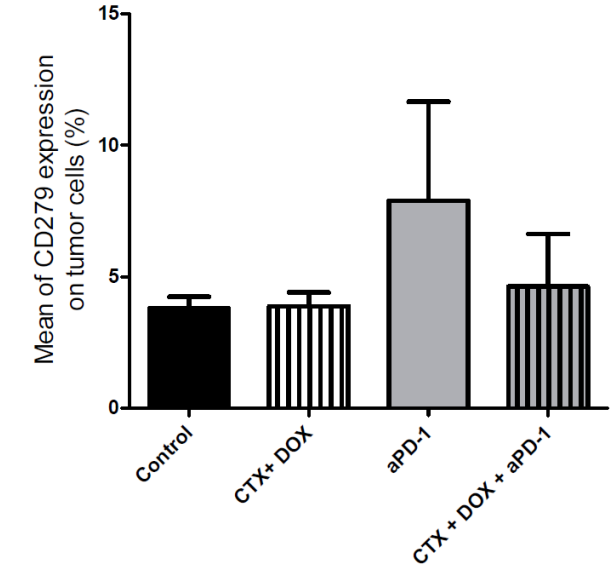

**D**

**PDL-1**

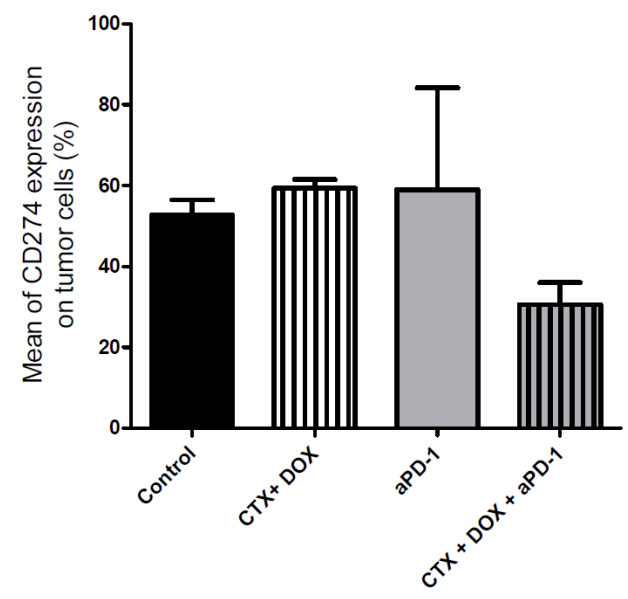

**E**

**TIGIT**

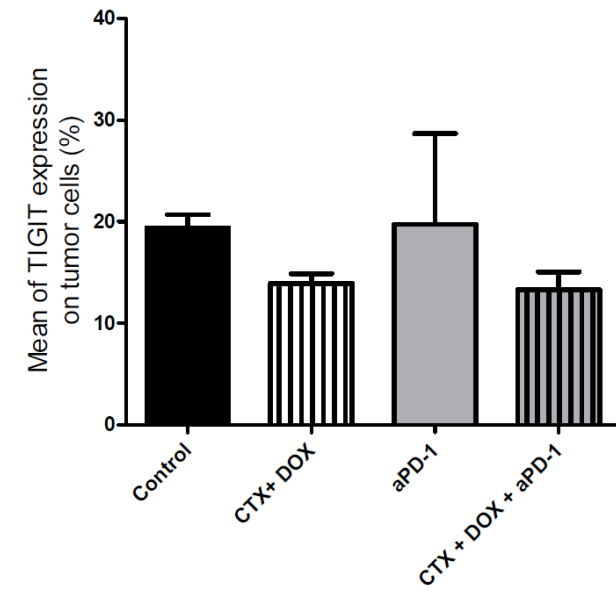

**F**

**TIM-3**

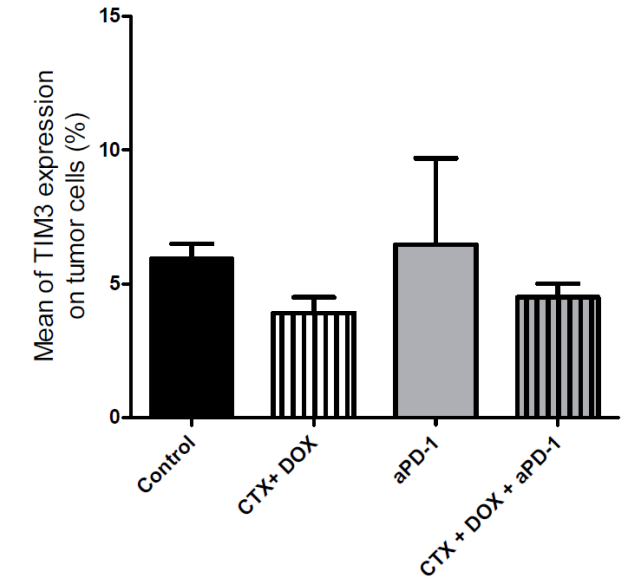

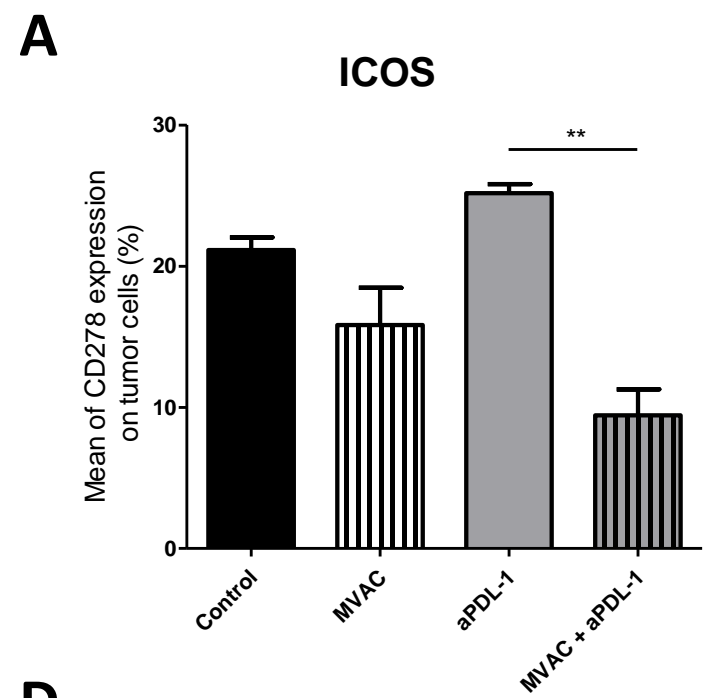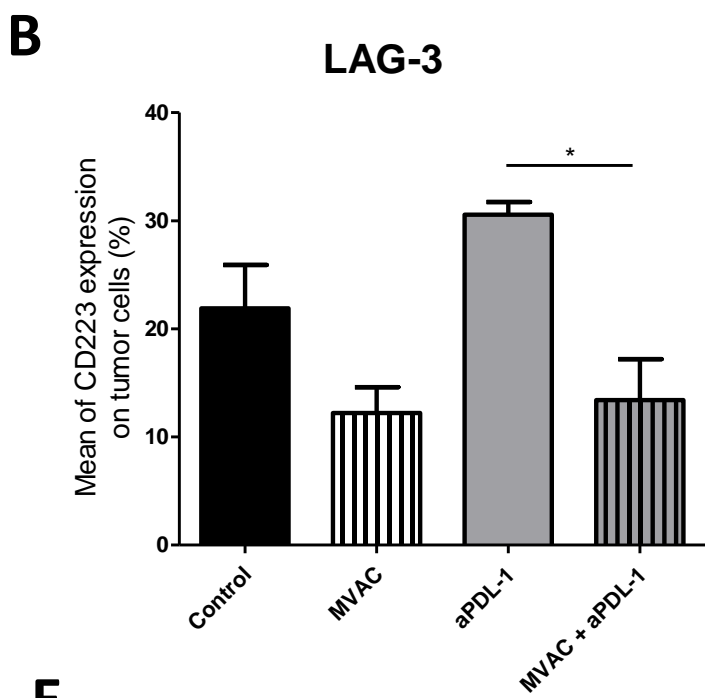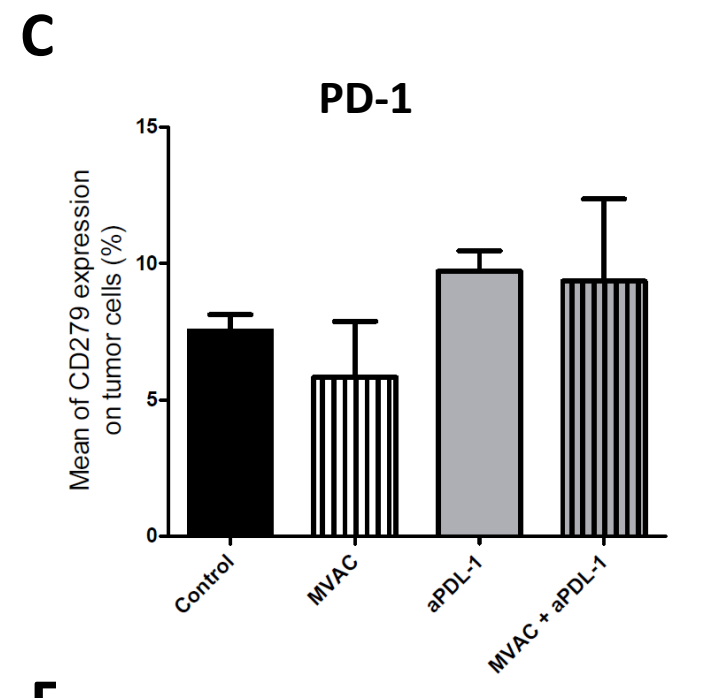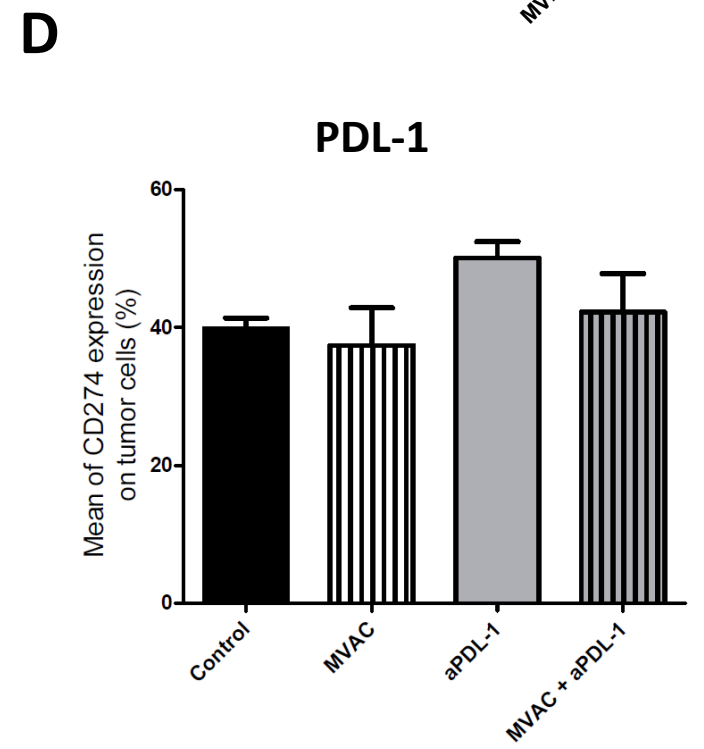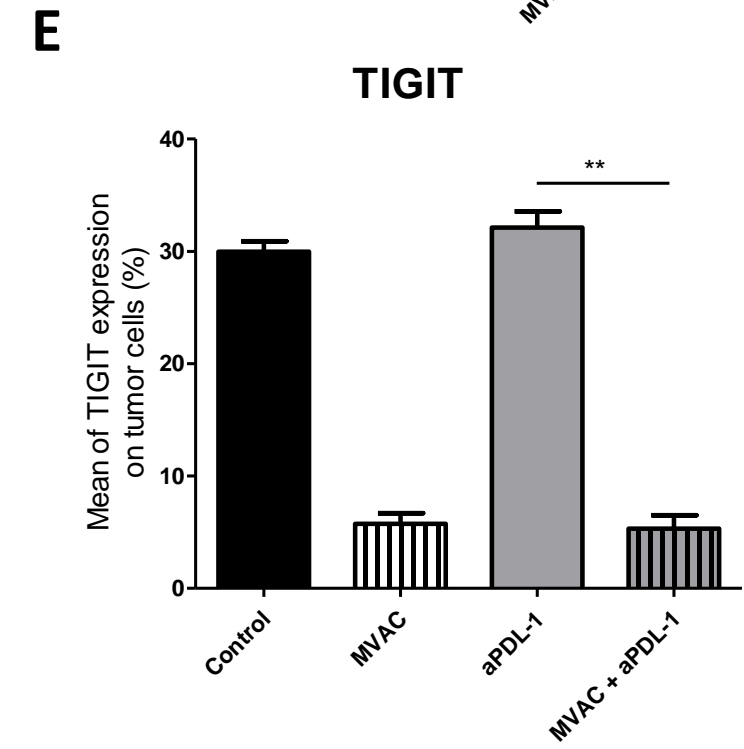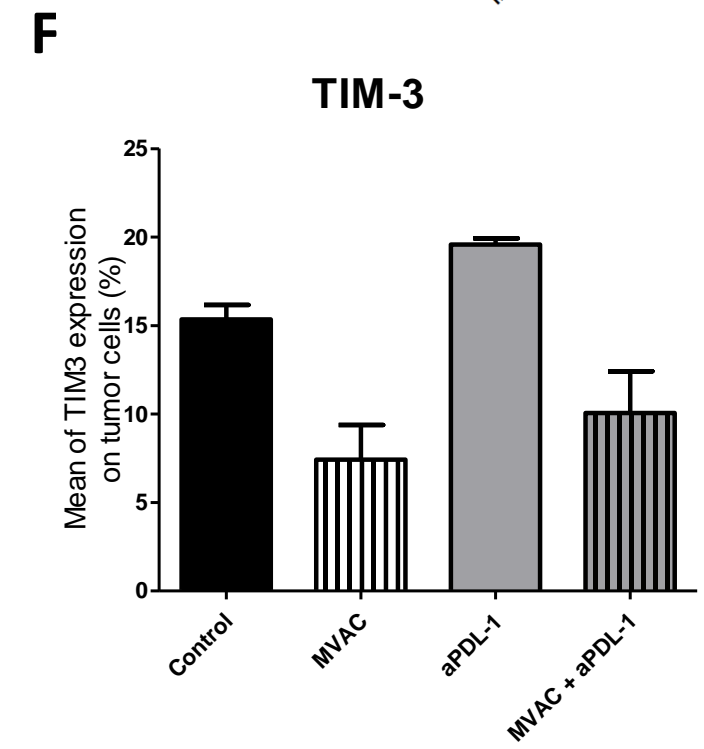

**A**

**ICOS**

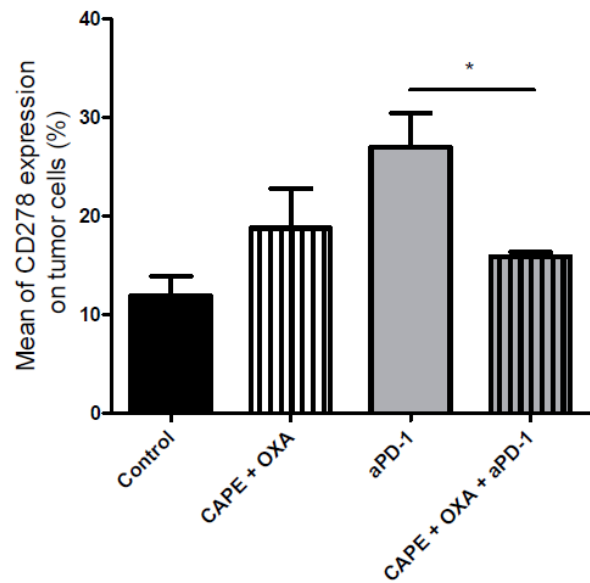

**B**

**LAG-3**

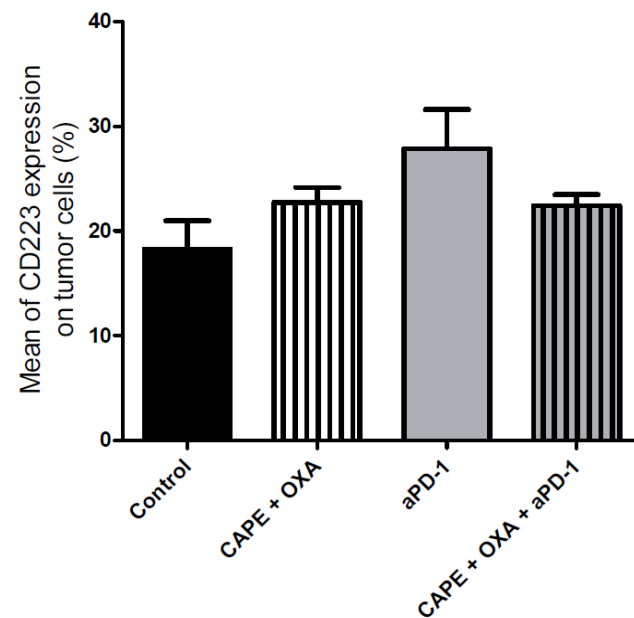

**C**

**PD-1**

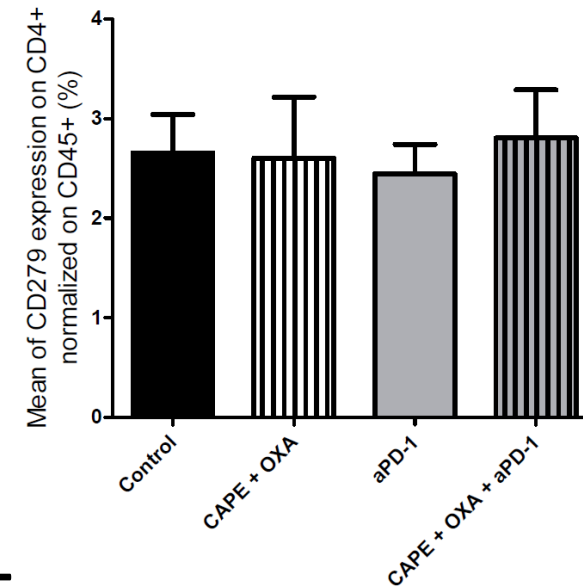

**D**

**PDL-1**

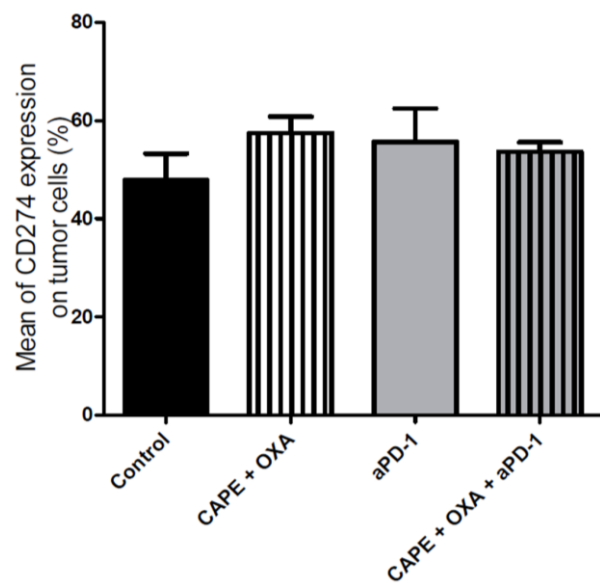

**E**

**TIGIT**

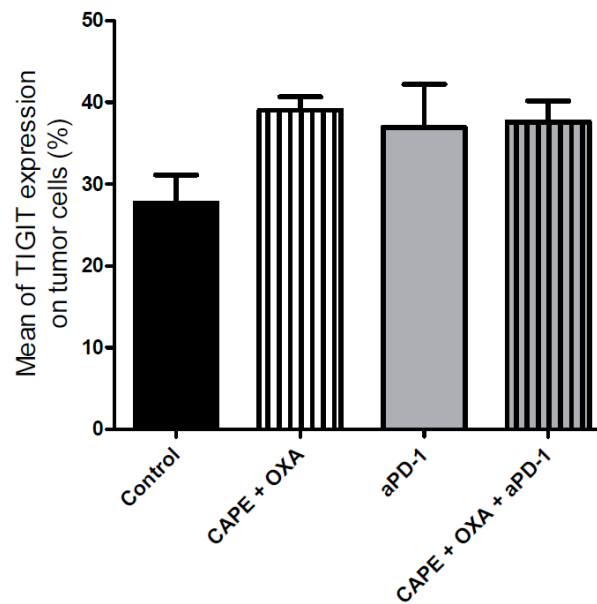

**F**

**TIM-3**

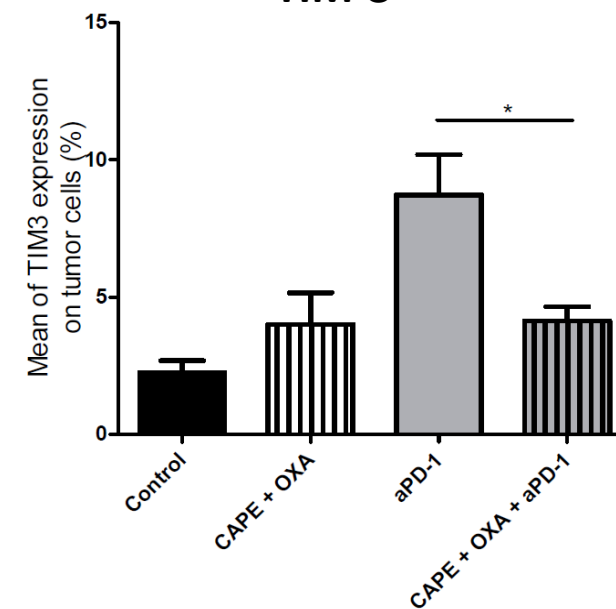

Supplement: Figure S8 — Effect of chemotherapies, anti-PD1 or anti-PDL1 Mabs and their combination on alternative immune checkpoints expression on tumor cells in MBT-2, 4T1, MB49 and MC38 preclinical tumor models. Flow cytometric analysis of CD278 (ICOS) (A), CD223 (LAG-3) (B), CD279 (PD-1) (C), CD274 (PDL-1) (D), TIGIT (E) and TIM-3 (F) on tumor cells infiltrate (CD45- cells). Mice were treated as in Table S1. Data are shown as mean values+ SEM, n = 5 to 6 mice/group (A), n = 5 to 6 mice/group (B), n = 6 mice/group (C), n = 5 mice/group (D). *p < 0.05, **p < 0.01, and ***p < 0.001 using Mann-Whitney test. [file Image_8.PDF]
