# Supplementary material for: The Antitumor Activity of Combinations of Cytotoxic Chemotherapy and Immune Checkpoint Inhibitors Is Model-Dependent
Source: Front Immunol. 2018 Oct 9;9:2100. doi: 10.3389/fimmu.2018.02100 (PMC6190749; doi:10.3389/fimmu.2018.02100)
Supplement: Figure S6 — Effect of chemotherapies, anti-PD1 or anti-PDL1 Mabs and their combination on alternative immune checkpoints expression on CD8+ T cells in MBT-2, 4T1, MB49 and MC38 preclinical tumor models. Flow cytometric analysis of CD278 (ICOS) (A), CD223 (LAG-3) (B), CD279 (PD-1) (C), CD274 (PDL-1) (D), TIGIT (E), and TIM-3 (F) on CD8+ T cells infiltrate of CD45+ cells. Mice were treated as in Table S1. Data are shown as mean values+ SEM, n = 5 to 6 mice/group (A), n = 5 to 6 mice/group (B), n = 6 mice/group (C), n = 5 mice/group (D). [file Image_6.PDF]

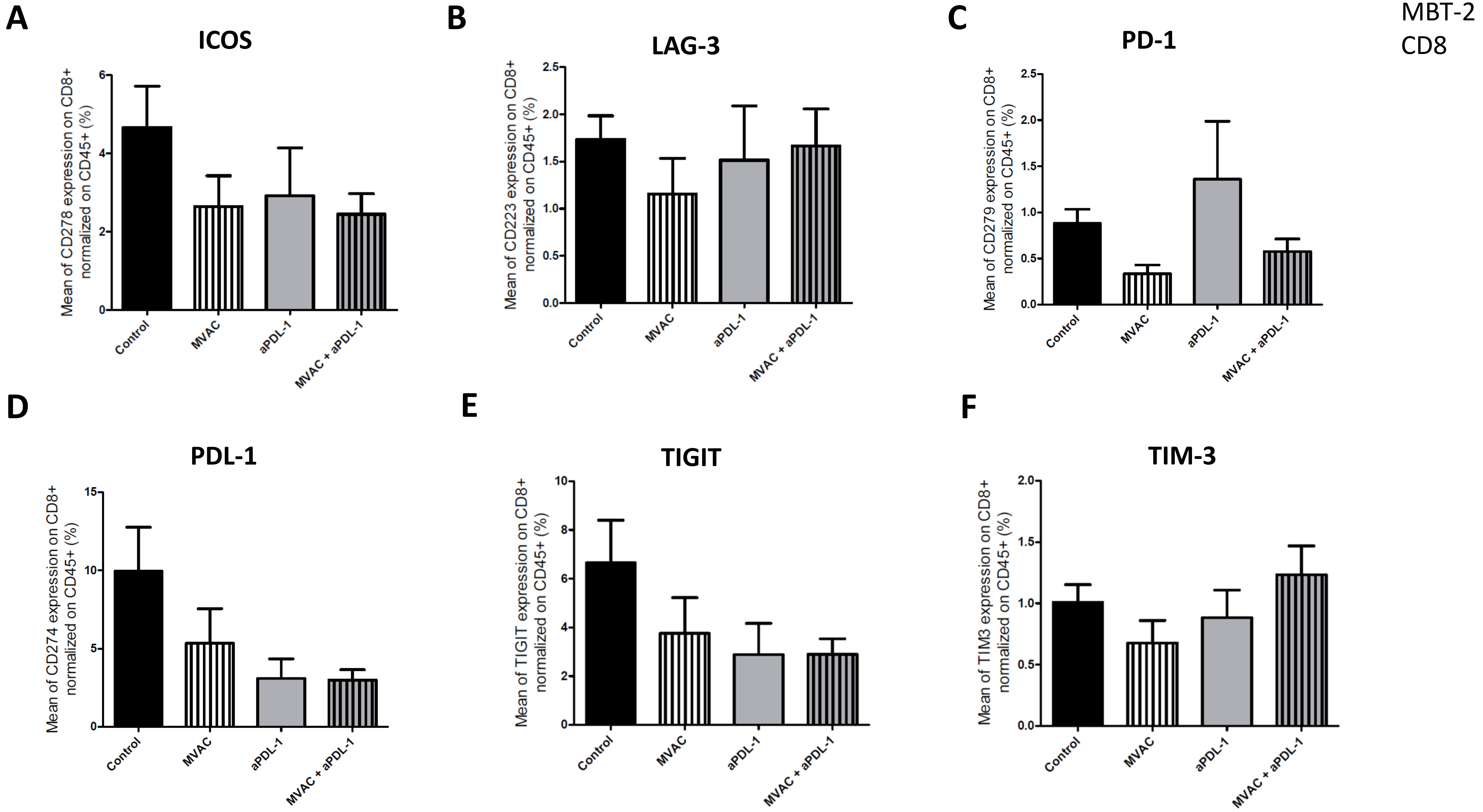
MBT-2
CD8
A
ICOS
Mean of CD278 expression on CD8+ normalized on CD45+ (%)
0
2
4
6
Control
MVAC
aPDL-1
MVAC + aPDL-1
B
LAG-3
Mean of CD223 expression on CD8+ normalized on CD45+ (%)
0.0
0.5
1.0
1.5
2.0
2.5
Control
MVAC
aPDL-1
MVAC + aPDL-1
C
PD-1
Mean of CD279 expression on CD8+ normalized on CD45+ (%)
0.0
0.5
1.0
1.5
2.0
2.5
Control
MVAC
aPDL-1
MVAC + aPDL-1
D
PDL-1
Mean of CD274 expression on CD8+ normalized on CD45+ (%)
0
5
10
15
Control
MVAC
aPDL-1
MVAC + aPDL-1
E
TIGIT
Mean of TIGIT expression on CD8+ normalized on CD45+ (%)
0
2
4
6
8
10
Control
MVAC
aPDL-1
MVAC + aPDL-1
F
TIM-3
Mean of TIM3 expression on CD8+ normalized on CD45+ (%)
0.0
0.5
1.0
1.5
2.0
Control
MVAC
aPDL-1
MVAC + aPDL-1

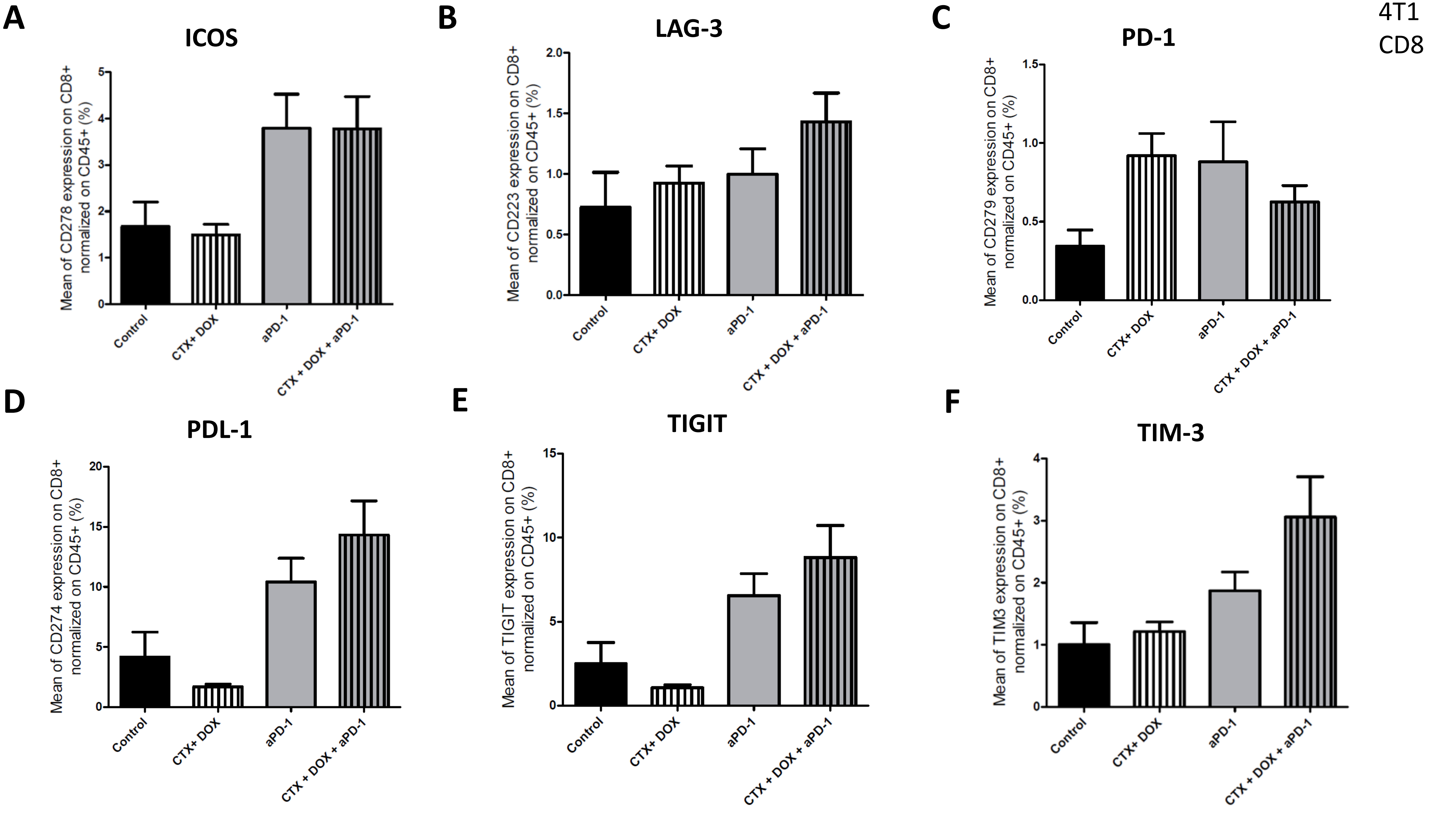
4T1
CD8
A
ICOS
Mean of CD278 expression on CD8+ normalized on CD45+ (%)
0
1
2
3
4
5
Control
CTX+ DOX
aPD-1
CTX + DOX + aPD-1
B
LAG-3
Mean of CD223 expression on CD8+ normalized on CD45+ (%)
0.0
0.5
1.0
1.5
2.0
Control
CTX+ DOX
aPD-1
CTX + DOX + aPD-1
C
PD-1
Mean of CD279 expression on CD8+ normalized on CD45+ (%)
0.0
0.5
1.0
1.5
Control
CTX+ DOX
aPD-1
CTX + DOX + aPD-1
D
PDL-1
Mean of CD274 expression on CD8+ normalized on CD45+ (%)
0
5
10
15
20
Control
CTX+ DOX
aPD-1
CTX + DOX + aPD-1
E
TIGIT
Mean of TIGIT expression on CD8+ normalized on CD45+ (%)
0
5
10
15
Control
CTX+ DOX
aPD-1
CTX + DOX + aPD-1
F
TIM-3
Mean of TIM3 expression on CD8+ normalized on CD45+ (%)
0
1
2
3
4
Control
CTX+ DOX
aPD-1
CTX + DOX + aPD-1

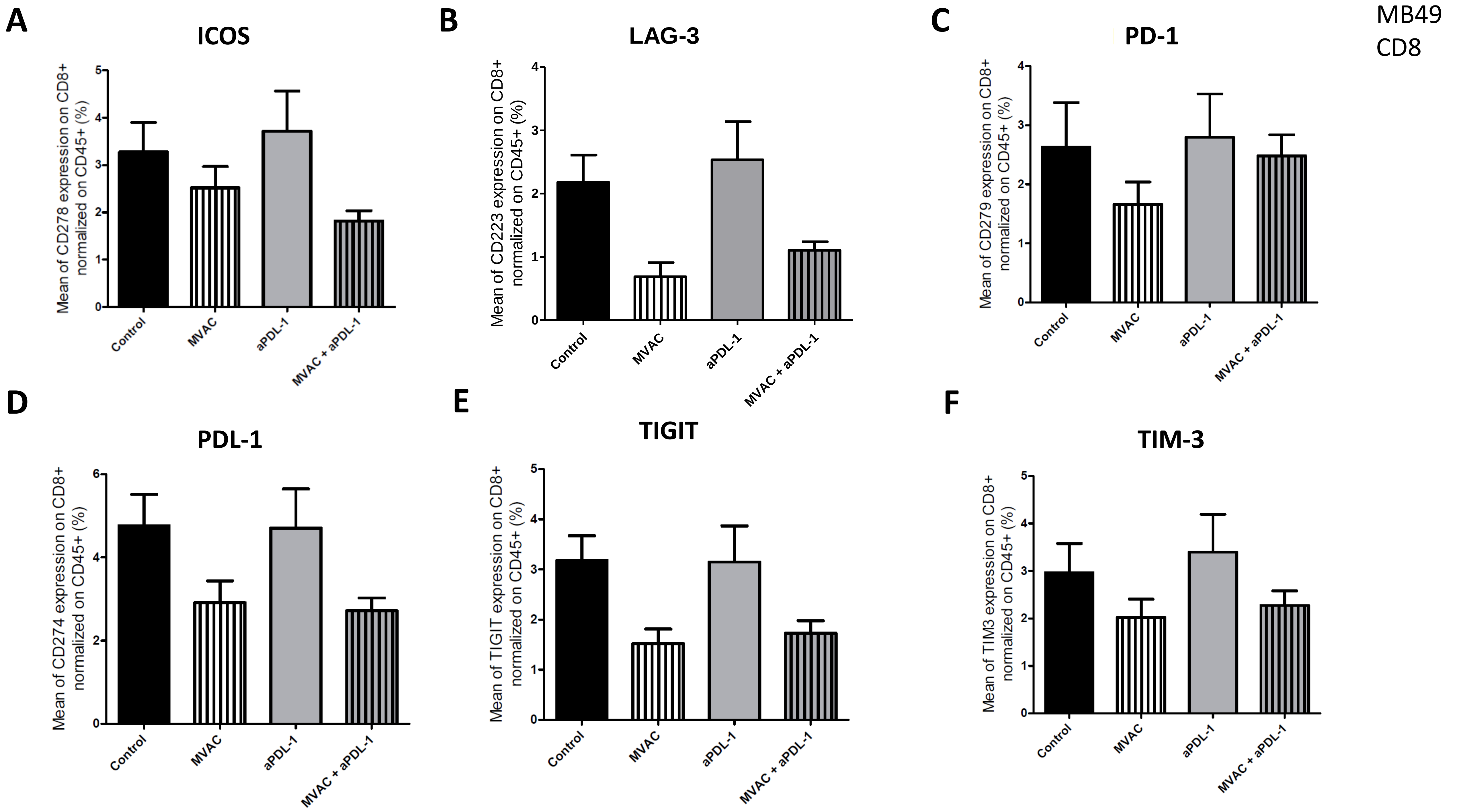
MB49
CD8
A
ICOS
Mean of CD278 expression on CD8+ normalized on CD45+ (%)
B
LAG-3
Mean of CD223 expression on CD8+ normalized on CD45+ (%)
C
PD-1
Mean of CD279 expression on CD8+ normalized on CD45+ (%)
D
PDL-1
Mean of CD274 expression on CD8+ normalized on CD45+ (%)
E
TIGIT
Mean of TIGIT expression on CD8+ normalized on CD45+ (%)
F
TIM-3
Mean of TIM3 expression on CD8+ normalized on CD45+ (%)
Control
MVAC
aPDL-1
MVAC + aPDL-1

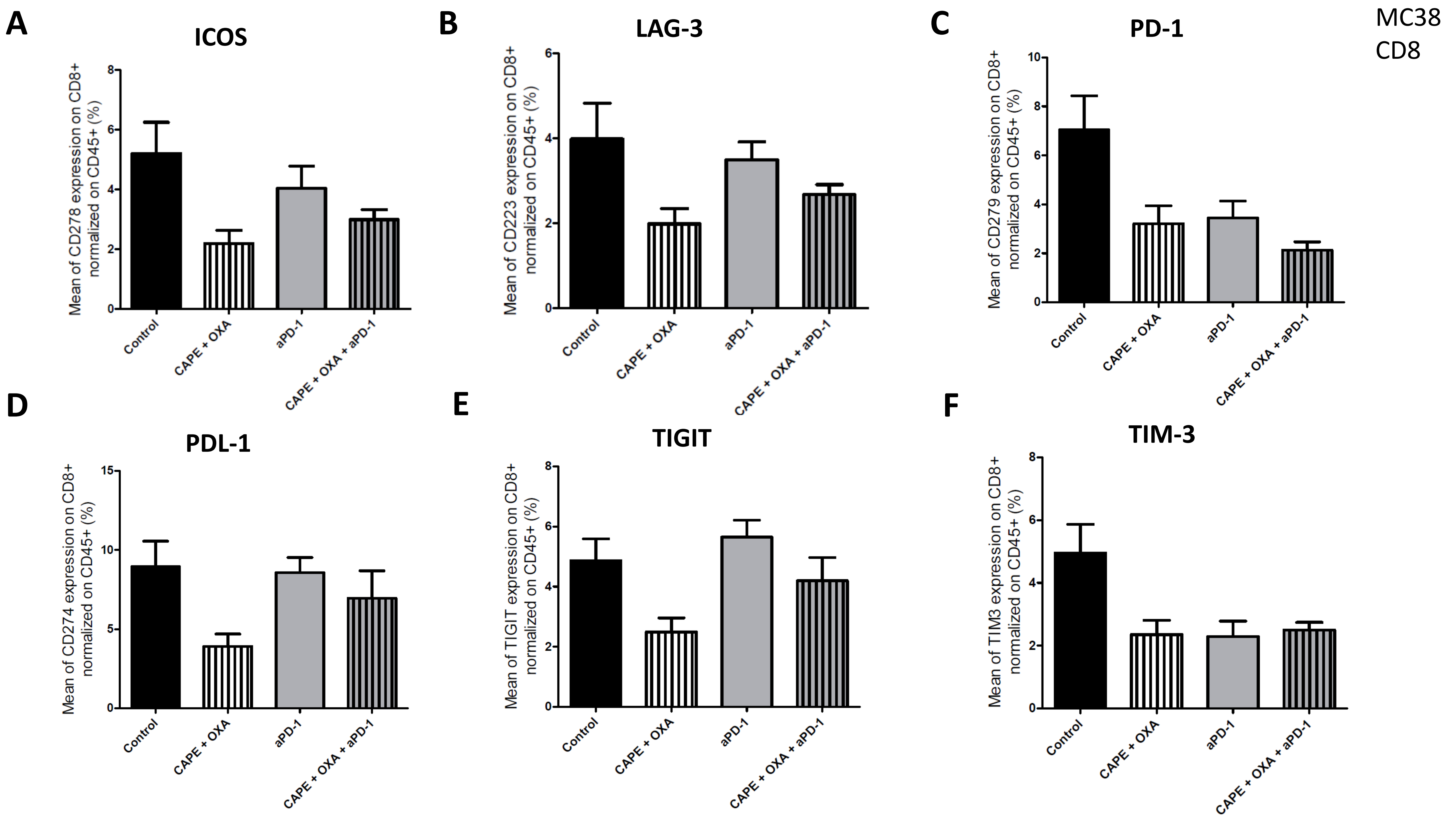

MC38
CD8
A
ICOS
Mean of CD278 expression on CD8+ normalized on CD45+ (%)
0
2
4
6
8
Control
CAPE + OXA
aPD-1
CAPE + OXA + aPD-1
B
LAG-3
Mean of CD223 expression on CD8+ normalized on CD45+ (%)
0
2
4
6
Control
CAPE + OXA
aPD-1
CAPE + OXA + aPD-1
C
PD-1
Mean of CD279 expression on CD8+ normalized on CD45+ (%)
0
2
4
6
8
10
Control
CAPE + OXA
aPD-1
CAPE + OXA + aPD-1
D
PDL-1
Mean of CD274 expression on CD8+ normalized on CD45+ (%)
0
5
10
15
Control
CAPE + OXA
aPD-1
CAPE + OXA + aPD-1
E
TIGIT
Mean of TIGIT expression on CD8+ normalized on CD45+ (%)
0
2
4
6
8
Control
CAPE + OXA
aPD-1
CAPE + OXA + aPD-1
F
TIM-3
Mean of TIM3 expression on CD8+ normalized on CD45+ (%)
0
2
4
6
8
Control
CAPE + OXA
aPD-1
CAPE + OXA + aPD-1
