# Supplementary material for: The Antitumor Activity of Combinations of Cytotoxic Chemotherapy and Immune Checkpoint Inhibitors Is Model-Dependent
Source: Front Immunol. 2018 Oct 9;9:2100. doi: 10.3389/fimmu.2018.02100 (PMC6190749; doi:10.3389/fimmu.2018.02100)
Supplement: Table S1 — In vivo experiments design overview. [file Data_Sheet_1.docx]

**Supplementary tables:**

| **Tumor type** | **Group** | **Treatment** | **Dose (mg/kg)** | **Route** | **Treatment schedule** |
| --- | --- | --- | --- | --- | --- |
| Colorectal cancer MC38 | 1 | Non treated (control) | - | - | - |
|  | 2 | anti-PD-1 | 12.5 | IP | 1/ week |
|  | 3 | capecitabine | 250 | Per Os | 5 days/week |
|  |  | oxaliplatin | 5 | IP | 1/week |
|  | 4 | capecitabine | 250 | Per Os | 5 days/week |
|  |  | oxaliplatin | 5 | IP | 1/week |
|  |  | anti-PD-1 | 12.5 | IP | 1/ week |
| Bladder cancer MB49 | 1 | Non treated (control) | - | - | - |
|  | 2 | anti-PD-1 | 12.5 | IP | 1/ week |
|  | 3 | anti-PD-L1 | 12.5 | IP | 1/ week |
|  | 4 | methotrexate | 1 | IP | 1/week |
|  |  | vinblastine | 0.1 | IP | 1/week |
|  |  | doxorubicin | 1 | IP | 1/week |
|  |  | cisplatin | 1 | IP | 1/week |
|  | 5 | methotrexate | 1 | IP | 1/week |
|  |  | vinblastine | 0.1 | IP | 1/week |
|  |  | doxorubicin | 1 | IP | 1/week |
|  |  | cisplatin | 1 | IP | 1/week |
|  |  | anti-PD-1 | 12.5 | IP | 1/ week |
|  | 6 | methotrexate | 1 | IP | 1/week |
|  |  | vinblastine | 0.1 | IP | 1/week |
|  |  | doxorubicin | 1 | IP | 1/week |
|  |  | cisplatin | 1 | IP | 1/week |
|  |  | anti-PD-L1 | 12.5 | IP | 1/ week |
| Metastatic breast cancer 4T1 | 1 | Non treated (control) | - | - | - |
|  | 2 | anti-PD-1 | 12.5 | IP | 1/ week |
|  | 3 | anti-PD-L1 | 12.5 | IP | 1/ week |
|  | 4 | cyclophosphamide | 100 | IP | 1/ week |
|  |  | doxorubicin | 2 | IP | 1/week |
|  | 5 | cyclophosphamide | 100 | IP | 1/ week |
|  |  | doxorubicin | 2 | IP | 1/week |
|  |  | anti-PD-1 | 12.5 | IP | 1/ week |
|  | 6 | cyclophosphamide | 100 | IP | 1/ week |
|  |  | doxorubicin | 2 | IP | 1/week |
|  |  | anti-PD-L1 | 12.5 | IP | 1/ week |
| Bladder cancer MB49 | 1 | Non treated (control) | - | - | - |
|  | 2 | anti-PD-L1 | 12.5 | IP | 1/ week |
|  | 3 | methotrexate | 1 | IP | 1/week |
|  |  | vinblastine | 0.1 | IP | 1/week |
|  |  | doxorubicin | 1 | IP | 1/week |
|  |  | cisplatin | 1 | IP | 1/week |
|  | 4 | methotrexate | 1 | IP | 1/week |
|  |  | vinblastine | 0.1 | IP | 1/week |
|  |  | doxorubicin | 1 | IP | 1/week |
|  |  | cisplatin | 1 | IP | 1/week |
|  |  | anti-PD-L1 | 12.5 | IP | 1/ week |

**Table S1**. In vivo experiments design overview

| **Tube 1** | |
| --- | --- |
| **Population** | **Label** |
| Teff | CD45+ CD3+ CD4- CD8+ TNα+ |
| Treg | CD45+ CD3+ CD4+ CD8- FoxP3+ |
| **Tube 2** | |
| **Population** | **Label** |
| Total MDSC | CD45+ CD3- CD11b+ |
| M-MDSC | CD45+ CD3- CD11b+ Ly6G- Ly6C+ |
| G-MDSC | CD45+ CD3- CD11b+ Ly6G+ Ly6C- |
| Total TAM | CD45+ CD3- CD11b+ |
| M1 TAM | CD45+ CD3- CD11b+ CD68+ CD206- |
| M2 TAM | CD45+ CD3- CD11b+ CD68- CD206+ |
| **Tube 3** | |
| **Population** | **Label** |
| PD-1 | CD45+ CD3+ CD4+/CD8+ CD279+ ; CD45- CD279+ |
| LAG-3 | CD45+ CD3+ CD4+/CD8+ CD223+; CD45- CD223+ |
| TIM-3 | CD45+ CD3+ CD4+/CD8+ TIM-3+; CD45- TIM-3+ |
| ICOS | CD45+ CD3+ CD4+/CD8+ CD278+; CD45- CD278+ |
| PDL-1 | CD45+ CD3+ CD4+/CD8+ CD274+; CD45- CD274+ |
| TIGIT | CD45+ CD3+ CD4+/CD8+ TIGIT+; CD45- TIGIT+ |

**Table S2**. Flow cytometry assay design overview

|  | **ICI alone** | **ICI in combination with reference treatment** |
| --- | --- | --- |
| **Bladder cancer** | 7 | 17 |
| **Colorectal cancer** | 8 | 35 |
| **Breast cancer** | 3 | 41 |

**Table S3**. Current Phase I-II clinical trials using anti-PD-1 or anti-PDL-1 in breast, colorectal and bladder cancer (from [www.clinicaltrials.gov](http://www.clinicaltrials.gov), searched items “nivolumab” and “atezolizumab”, 2018, ICI= Immune Checkpoint Inhibitor)
